# *Chlamydia* exploits filopodial capture and a macropinocytosis-like pathway for host cell entry

**DOI:** 10.1371/journal.ppat.1007051

**Published:** 2018-05-04

**Authors:** Charlotte Ford, Andrea Nans, Emmanuel Boucrot, Richard D. Hayward

**Affiliations:** 1 Institute of Structural and Molecular Biology, Birkbeck & University College London, London, United Kingdom; 2 Institute of Structural and Molecular Biology, University College London & Birkbeck, London, United Kingdom; University of California, Berkeley, UNITED STATES

## Abstract

Pathogens hijack host endocytic pathways to force their own entry into eukaryotic target cells. Many bacteria either exploit receptor-mediated zippering or inject virulence proteins directly to trigger membrane reorganisation and cytoskeletal rearrangements. By contrast, extracellular *C*. *trachomatis* elementary bodies (EBs) apparently employ facets of both the zipper and trigger mechanisms and are only ~400 nm in diameter. Our cryo-electron tomography of *C*. *trachomatis* entry revealed an unexpectedly diverse array of host structures in association with invading EBs, suggesting internalisation may progress by multiple, potentially redundant routes or several sequential events within a single pathway. Here we performed quantitative analysis of actin organisation at chlamydial entry foci, highlighting filopodial capture and phagocytic cups as dominant and conserved morphological structures early during internalisation. We applied inhibitor-based screening and employed reporters to systematically assay and visualise the spatio-temporal contribution of diverse endocytic signalling mediators to *C*. *trachomatis* entry. In addition to the recognised roles of the Rac1 GTPase and its associated nucleation-promoting factor (NPF) WAVE, our data revealed an additional unrecognised pathway sharing key hallmarks of macropinocytosis: i) amiloride sensitivity, ii) fluid-phase uptake, iii) recruitment and activity of the NPF N-WASP, and iv) the localised generation of phosphoinositide-3-phosphate (PI3P) species. Given their central role in macropinocytosis and affinity for PI3P, we assessed the role of SNX-PX-BAR family proteins. Strikingly, SNX9 was specifically and transiently enriched at *C*. *trachomatis* entry foci. SNX9^-/-^ cells exhibited a 20% defect in EB entry, which was enhanced to 60% when the cells were infected without sedimentation-induced EB adhesion, consistent with a defect in initial EB-host interaction. Correspondingly, filopodial capture of *C*. *trachomatis* EBs was specifically attenuated in SNX9^-/-^ cells, implicating SNX9 as a central host mediator of filopodial capture early during chlamydial entry. Our findings identify an unanticipated complexity of signalling underpinning cell entry by this major human pathogen, and suggest intriguing parallels with viral entry mechanisms.

## Author summary

*Chlamydia trachomatis* remains the leading bacterial agent of sexually transmitted disease worldwide and causes a form of blindness called trachoma in Developing nations, which is recognised by the World Health Organisation as a neglected tropical disease. Despite this burden, we know comparatively little about how it causes disease at a molecular level. *Chlamydia* must live inside human cells to survive, and here we study the mechanism of how it enters cells, which is critical to the lifecycle. We study how the bacterium exploits signalling pathways inside the cell to its own advantage to deform the cell membrane by reorganising the underlying cell skeleton, and identify new factors involved in this process. Our findings suggest intriguing similarities with how some viruses enter cells. A better understanding of these processes may help to develop future vaccines and new treatments.

## Introduction

An essential early event in the lifecycle of many human and animal pathogens is entry into non-phagocytic host epithelial cells. Viruses, bacteria and parasites all engage with host cell surfaces prior to inducing the reorganisation of the plasma membrane and underlying cytoskeleton to promote their internalisation. Invasive bacteria like *Salmonella* and *Listeria* species are typically > 1 μm in diameter and promote their internalisation either by injecting virulence effector proteins that subvert host signalling to reversibly induce cytoskeletal reorganisation, or through surface ligand mimicry hijack receptor-mediated endocytosis, respectively [1]. By contrast, the infectious extracellular form of *Chlamydia trachomatis*, termed the elementary body (EB), is much smaller in diameter (~400 nm) than its archetypal Gram-negative cousins. Nevertheless, EBs must also induce actin-dependent internalisation into non-phagocytic cells, a pivotal step in the lifecycle of this obligate intracellular bacterium [2].

How *C*. *trachomatis* promotes cell entry is incompletely understood, but it is often considered as an example of the trigger mechanism epitomised by the enteroinvasive bacterium *Salmonella typhimurium*. *Salmonella* employs a type III secretion system (T3SS) to inject multiple, semi-redundant effectors into host cells that coordinate the reorganisation of the host actin cytoskeleton. Two effectors reversibly stimulate the cellular Rho-family GTPases Cdc42 and Rac1, two effectors bind to actin directly to modulate filament dynamics, and a further effector acts as a phosphoinositol phosphatase mimic, modulating membrane plasticity and co-stimulating the Rho GTPases [3–7]. *C*. *trachomatis* EBs also exploit a T3SS and deliver effectors into the host cell that reversibly stimulate Rac1. Although the mechanism remains incomplete, a major factor is the T3SS effector translocated actin recruiting phosphoprotein (TARP) that nucleates polymerisation directly by binding to actin, and indirectly upon tyrosine phosphorylation by acting as a scaffold for Rac1 guanine nucleotide exchange factors [8–11]. A second effector post-translationally modifies the GTPase itself, possibly to subsequently downregulate signaling [12]. A number of host receptors have also been linked to cell entry by different chlamydial species. For instance, *C*. *pneumoniae* Pmp21 binds epidermal growth factor receptor (EGFR) to induce EB entry by receptor-mediated endocytosis [13], an event more reminiscent of the zipper mechanism exemplified by *Listeria* [14]. The role of receptors in *C*. *trachomatis* entry is however less clear, as none are essential [15].

Although Rac1 stimulation is sufficient for the formation of lamellipodia, this signalling activity cannot exclusively account for the complex actin ruffles, pedestal-like structures and filopodia present at EB entry sites [2,16]. This view was further reinforced by our cryo-electron tomography of early interactions between *C*. *trachomatis* EBs and cultured cells [17], when EBs were captured in association with phagocytic cups, trapped by actin-rich filopodia and present within membrane ruffles reminiscent of macropinosomes. These combined data support a view that multiple or redundant entry pathways are likely to operate in parallel. As expected, chlamydial entry thus shares many similarities with other bacterial entry pathways. However, there are also striking parallels with viral entry mechanisms. The small size of EBs, their association with filopodia [2], entry-associated phosphorylation and signaling [16,18,19], and also the requirement for protein disulphide isomerase-associated reduction [20] and promiscuous receptor interactions [21–23] are all factors common to viral entry mechanisms [24–28]. In this study we have quantified the cytoskeletal rearrangements and membrane reorganisation at *C*. *trachomatis* entry foci, and systematically investigated the underlying signalling pathways, initially by employing inhibitor screens in a manner analogous to studies of viral entry [29,30].

## Results

### Filopodial association is an early event during *C*. *trachomatis* entry

Although our cryo-electron tomography revealed an unexpected diversity of cellular structures at *C*. *trachomatis* entry sites [17], this technique did not enable the visualisation of sufficient events to statistically distinguish whether they represent sequential assemblies or distinct pathways. Consequently, we exploited confocal microscopy to categorise a significantly larger number of bacterial entry foci, initially by observing F-actin recruitment during *C*. *trachomatis* infection of human retinal pigment epithelial (RPE1) cells. Telomerase-immortalised RPE1 cells are widely applied to study endocytic pathways [31,32], and are permissive to *C*. *trachomatis* infection [33].

**Fig 1:**
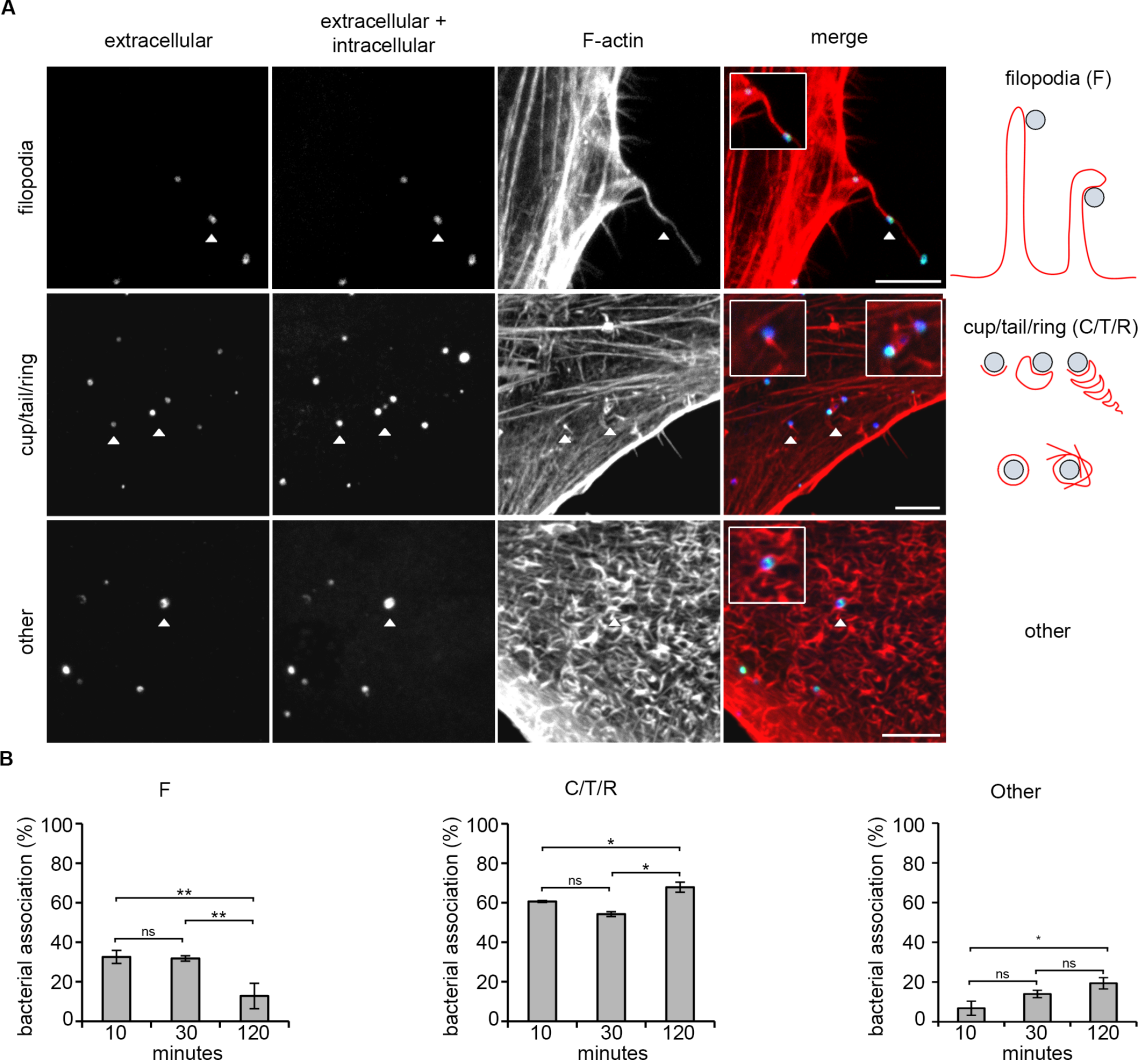
F-actin recruitment to *C*. *trachomatis* entry sites in cultured RPE1 cells. (A) Representative immunofluorescence images of F-actin recruitment to *C*. *trachomatis* EBs during early interaction with RPE1 cells. Cultured RPE1 cells were infected with *C*. *trachomatis* LGV2 for 30 minutes prior to fixation with 1% paraformaldehyde. Fixed cells were stained with an anti-*Chlamydia* primary antibody and an Alexa Fluor 488-conjugated secondary antibody. Cells were permeabilised with 0.05% Triton X-100 (v/v) and the bacteria stained using the same anti-*Chlamydia* primary antibody and an Alexa Fluor 633-conjugated secondary antibody. Intracellular bacteria were labelled with only Alexa Fluor 633 (dark blue; ‘intracellular and extracellular’ panel), extracellular bacteria were labelled with Alexa Fluor 488 and Alexa Fluor 633 (green + blue, cyan; ‘extracellular’ and ‘intracellular and extracellular’ panels). F-actin was stained with rhodamine-phalloidin. White arrowheads show typical examples of indicated classes of F-actin structure. Images are maximum projections of confocal xy sections. Scale bars, 5 μm. Right hand panels show diagrammatic representations of the defined classes of F-actin structures visualised by fluorescence microscopy of cultured RPE1 cells infected with *C*. *trachomatis*. (B) Quantification of F-actin structures associated with extracellular *C*. *trachomatis* EBs during infection of RPE1 cells. Cells were infected for 10, 30 and 120 min prior to fixation with 1% PFA. Fixed cells were stained as above and the association of EBs with the defined F-actin classes quantified. ≥ 200 bacteria were assessed at each time point and the percentage of EBs in association with each class of structure was calculated, expressed as the average ±SD (n = 3). * P<0.05, ** P<0.01 using one-way ANOVA followed by a Tukey's *post hoc* test.

Distinct F-actin structures could be defined using light microscopy that correlated with those observed by cryo-electron tomography [17]. Differential fluorescence staining was used to discriminate extracellular and intracellular bacteria (**S1 Fig, see Materials and Methods**). From 10 minutes post-infection, *C*. *trachomatis* LGV2 EBs were captured in association with filopodia, F-actin cup, tail or ring-like assemblies (**Fig 1A and additional examples in S2 Fig**). To examine the progression of these events over time, cells were additionally fixed 30 and 120 minutes post-infection. The most striking phenotype was the difference in the number of EBs in association with filopodia, which decreased from 33 ± 3% at 10 minutes to 13 ± 6% after 120 minutes (**Fig 1B**). This revealed that filopodial association is a significant early event during EB entry into cultured RPE1 cells.

To determine whether filopodial association is a conserved early event, F-actin structures were next equivalently quantified following the infection of HeLa cells with *C*. *trachomatis* LGV2 (**S3A Fig**). The frequency of filopodial association at 10 minutes post infection was even higher in these cells, at 51 ± 4% EBs, decreasing significantly by 120 minutes post infection to 23 ± 3% (**S3B Fig**). Comparable EB-associated F-actin structures formed when RPE1 cells were infected with *C*. *trachomatis* serovar D (**S4A and S4B Fig**). Under these experimental infection conditions, RPE1 and HeLa cells have similar overall infection kinetics, and comparable proportions of EBs in association with F-actin structures at 10, 30 and 120 minutes post-infection (**S5 Fig**). These quantitative data illustrate a spatio-temporal conservation in the F-actin superstructures formed during *C*. *trachomatis* entry, which occur independently of bacterial serovar and host cell type. Furthermore, they highlight filopodial association as a conserved and quantitatively significant early event during initial EB-host cell interaction. These initial data thus support a model that *Chlamydia* might utilise similar mechanisms to viruses and exosomes for entry into host cells, whereby filopodial capture and surfing precedes internalisation [24].

### Signalling diversity during *C*. *trachomatis* cell entry

Filopodia are induced by the activation of the GTPase Cdc42 and the associated actin nucleation-promoting factor (NPF) N-WASP [34,35], although additional efficiency factors are also required including IRTKS- and Ena/VASP-family proteins [36,37]. However, previous studies have shown Rac1-WAVE-Arp2/3 signalling is clearly required for *C*. *trachomatis* invasion, whereas Cdc42 is not [38,39]. To evaluate whether additional host factors are required for filopodial association and EB uptake, cells were infected in the presence of small molecule inhibitors targetting a broad panel of cellular factors known to influence actin dynamics and endocytic processes. Initially, to facilitate high-throughput screening, cultured RPE1 cells were infected with *C*. *trachomatis* LGV2 for two hours in the presence of three different concentrations of each inhibitor, where the mid concentration was the established IC_50_ or effective concentration. Inhibitors were then removed by washing, and the infection allowed to proceed until 24 hours post-infection (hpi), when the infected cells were fixed and the number of inclusions present in comparison to control, mock-treated cells enumerated. Based on the initial results, a second smaller-scale inhibitor screen was conducted to directly assess the effects of a subset of the inhibitors, which influenced inclusion formation in the first screen, on bacterial entry by applying differential fluorescence staining to discriminate extracellular and intracellular bacteria directly. This allowed effects on entry and nascent inclusion formation to be distinguished. For the initial screening at 24 hpi, effects were arbitrarily considered as significant when the mid-concentration of an inhibitor reduced the number of inclusion-containing cells to ≤ 75% of the control (this threshold is represented by a dotted line on plots in **S6 Fig**). Inclusion morphology was also examined following each treatment by parallel immunofluorescence (of which selected examples are shown in **S7 Fig**).

Consistent with the major roles for the Sos/Abi1/Eps8 and Vav2 guanine nucleotide exchange factors (GEFs) in the activation of the small GTPase Rac1 during *C*. *trachomatis* entry [11], the EHop inhibitor that specifically prevents Vav2-mediated Rac1 activation [40] induced a clear and significant dose-dependent decrease in the number of inclusion-containing cells, whereas NSC that alternatively targets Rac1-specific GEFs TrioN and Tiam1 had a lesser effect [41] (compare **EHop** and **NSC** in **S6 Fig**). Unexpectedly, inhibitors targeting the GTPase Cdc42 resulted in significant reduction in inclusion number (**S6 Fig**, **ML141** and **Casin**). Conversely, the inhibition of RhoA and Arf GTPases did not reduce inclusion formation to ≤ 75% of the control, our significance criteria (**S6 Fig**, **RhoA** and **Arf6**), in agreement with previous studies [38], although Arf6 activity has been implicated in the internalisation of the related *C*. *caviae* [42]. Finally, all three dynamin inhibitors tested decreased inclusion formation significantly and dose-dependently, with dynasore and MiTMAB preventing inclusion formation at the highest concentration (**S6 Fig, Dynasore, MiTMAB** and **OcTMAB**), in agreement with the established role of dynamin in mediating lipid transport early during inclusion biogenesis [43].

Despite the well-recognised requirement for actin reorganisation, equivalent assays performed using small molecule inhibitors that target actin dynamics exhibited contrasting effects on inclusion formation in RPE1 cells. Cytochalasin D treatment induced a dose-dependent decrease in inclusion formation, with an 88 ± 4% reduction at mid concentration (**S6 Fig, actin polymerisation cytoD**), whereas cells treated with latrunculin B only exhibited a 16 ± 7% decrease at the highest concentration tested (**S6 Fig, actin polymerisation latB**), despite the fact that both cytochalasin and latrunculin are classical inhibitors of actin polymerisation, albeit via distinct modes of action [44,45]. Jasplakinolide, which stabilises F-actin, prevented inclusion formation at the mid concentration (**S6 Fig, actin stabilisation**) [46]. Consistent with a role for Rho GTPase signalling, CK636 and CK548 that target the Arp2/3 actin nucleation complex and inhibit actin polymerisation either by preventing the Arp2 and Arp3 subunits of the complex entering their active conformation or by binding to the hydrophobic core of Arp3 [47], induced a dose-dependent reduction in inclusion formation, reflected by a 37 ± 8% and 48 ± 9% decreases at the mid concentration, respectively (**S6 Fig, CK636** and **CK548**). In addition to Arp2/3-directed nucleation of branched F-actin networks, unbranched filament nucleation by formins apparently also contributes, as the formin inhibitor SMIF induced a 76 ± 6% reduction in inclusion formation at the mid concentration, although this was excluded from further analysis as it did not reduce inclusion formation ≤ 75% of the control (**S6 Fig, SMIF**). These data demonstrate that our assays using *C*. *trachomatis* LGV2 and cultured RPE1 cells specifically recapitulate previous findings implicating the specific stimulation of Rac1 via a subfamily of cellular GEFs [11].

Given the early role of filopodia (**Fig 1**), we investigated the potential contribution of the NPF N-WASP, which stimulates filopodia formation by activating the Arp2/3 complex via Cdc42-dependent and -independent pathways [48,49]. We exploited wiskostatin that specifically inhibits N-WASP activity by stabilising the auto-inhibited conformation [50]. Intriguingly, N-WASP inhibition resulted in 49 ± 12% and 92 ± 5% decreases in inclusion formation at the mid-IC_50_ and high-concentrations, respectively (**S6 Fig, wiskostatin**). These data implicate N-WASP as an apparently dominant mediator of early inclusion formation. Correspondingly, the macropinocytosis inhibitor EIPA induced a dose-dependent decrease in inclusion formation (**S6 Fig, macropinocytosis**), with ~50% of the remaining inclusions containing RBs which were morphologically abnormal (**S7 Fig, EIPA**). By contrast, treatment with filipin and cholesterol oxidase, which target lipid raft and caveolae-mediated endocytosis [51,52], did not significantly affect the number of inclusions formed in comparison to the mock-treated controls (**S6 Fig, filipin & cholesterol oxidase**), or the morphology of the inclusions or RBs (**S7 Fig, cholesterol oxidase**). Tip, which targets myosin VI and consequently clathrin-mediated endocytosis (CME) [53], reduced inclusion formation by 15 ± 6% at the mid concentration, although the inhibitor itself was significantly cytotoxic when applied at higher concentrations (**S6 Fig, Tip** and **S8 Fig, Tip**). These initial inhibition experiments using inclusion formation 24 hpi as a phenotypic read-out in RPE1 cells, both confirmed recognised mediators of bacterial entry and early inclusion biogenesis (Rac1, dynamin, Arp2/3-dependent actin polymerisation) and implicated previously unrecognised factors (Cdc42, N-WASP, macropinocytosis).

**Fig 2:**
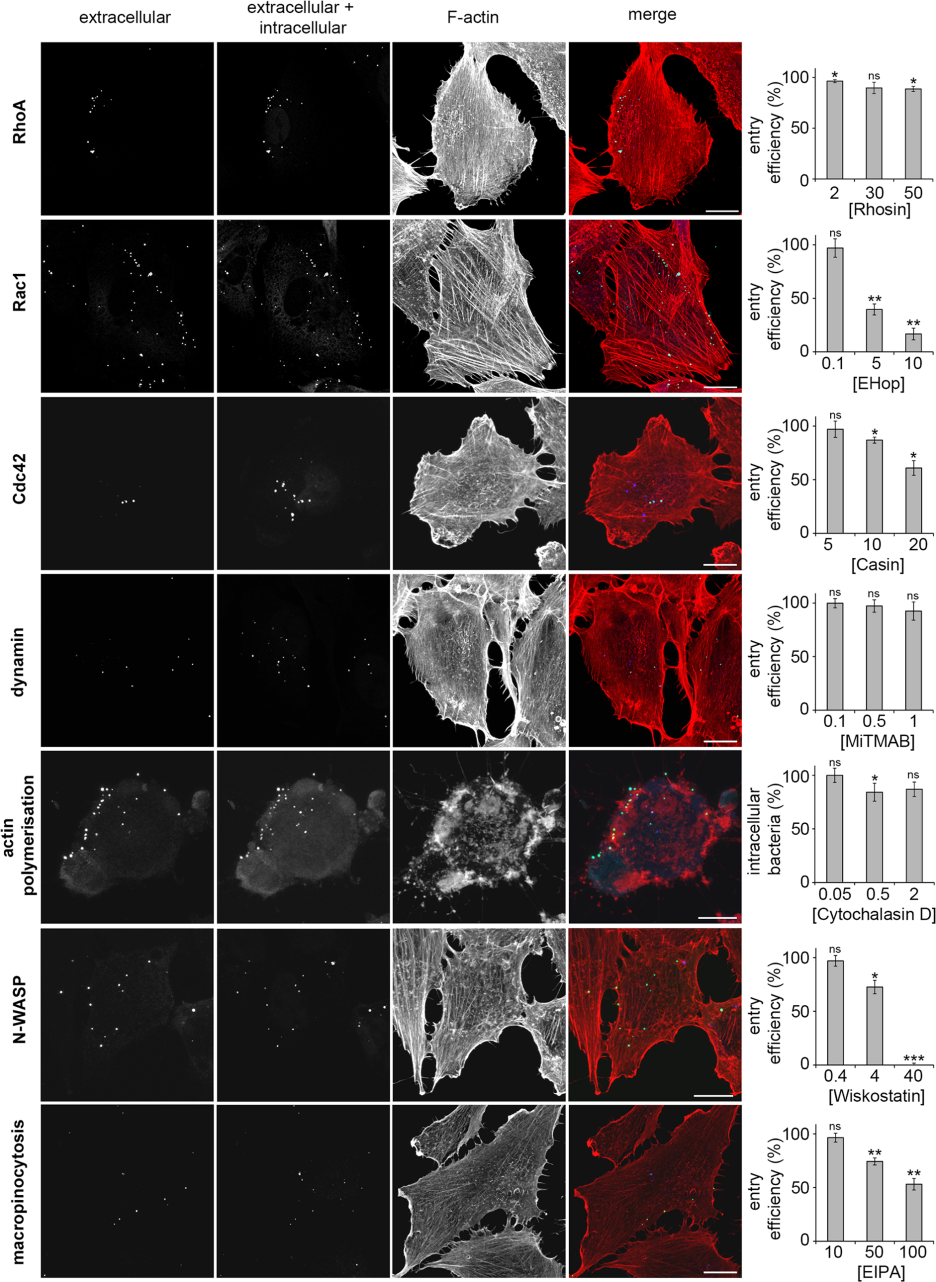
*C*. *trachomatis* invasion in the presence of small molecule inhibitors. Cultured RPE1 cells were pre-treated for 5 min with the indicated concentration of inhibitor, followed by infection with *Chlamydia trachomati*s LGV2 in the presence of inhibitor. At 2 h post-infection, cells were washed then fixed with 1% PFA and stained with an anti-*Chlamydia* primary antibody and an AlexaFluor 488-conjugated secondary antibody (green). Cells were permeablised and stained again using anti-*Chlamydia* primary antibody and an AlexaFluor 633-conjugated secondary antibody (blue). Intracellular bacteria were labelled with only one fluorophore (AlexaFluor 633). Images are maximum projections of confocal xy sections. Scale bars, 10 μm. The entry efficiency in the presence of inhibitors relative to mock treated control cells was quantified. ≥ 300 bacteria were assessed at each time point and the percentage entry efficiency of EBs is expressed as the average ±SD (n = 3). P-values obtained from Student's unpaired two-tailed t-test, * P<0.05, ** P<0.01, *** P<0.001, ‘ns’ not significant.

However, these data alone are insufficient to distinguish a role for these factors in *C*. *trachomatis* entry rather than in the subsequent development and trafficking of early bacteria-containing vacuoles. Based on the initial results (**S6 Fig**), a more restricted inhibitor screen was performed using EHop (Rac1), Casin (Cdc42), MiTMAB (dynamin), wiskostatin (N-WASP), CK636 (Arp2/3), cytochalasin D and latrunculin B (actin polymerisation), EIPA (macropinocytosis) and Rhosin (RhoA), to assess the effect of these inhibitors directly on bacterial entry using differential ‘inside-outside’ immunofluorescence staining (**S1 Fig**, see Materials and Methods). In agreement with their effects on inclusion formation (**S6 Fig** and **S7 Fig**), inhibition of Rac1, N-WASP, macropinocytosis and the Arp2/3 complex each reduced bacterial entry in a dose-dependent manner (**Fig 2** and **S9 Fig**). Indeed, the N-WASP inhibitor wiskostatin could block *C*. *trachomatis* entry, without inducing substantial cell cytotoxicity (**Fig 2, N-WASP** and **S8 Fig, Wiskostatin**). Consistent with the dominant role of Rac1 signalling [2,16], entry was not as profoundly suppressed by the Cdc42 inhibitor (compare **Rac1** and **Cdc42** in **Fig 2**), in agreement with previous studies [38,39]. Conversely, RhoA and dynamin GTPase inhibitors had a limited effect on *C*. *trachomatis* LGV2 entry (**Fig 2 RhoA** and **dynamin**), in agreement with a role for dynamin in mediating lipid transport post-entry, early during inclusion biogenesis [43]. Unexpectedly, neither cytochalasin D nor latrunculin B decreased bacterial entry as anticipated (**Fig 2, cytochalasin D** and **S9 Fig, latrunculin B**), although cytochalasin D treatment significantly inhibited the entry of *Salmonella typhimurium* into RPE1 cells, reducing internalisation to 18 ± 9% of mock-treated controls under equivalent conditions at IC_50_ (**S10A Fig**), when F-actin organisation is clearly disrupted (**S10B Fig**). Intriguingly, treatment with cytochalasin D or latrunculin B reproducibly stalled a population of EBs in association with patches of F-actin at the cell periphery (immunofluorescence panels in **Fig 2, cytochalasin D** and **S9 Fig, latrunculin B**).

**Fig 3:**
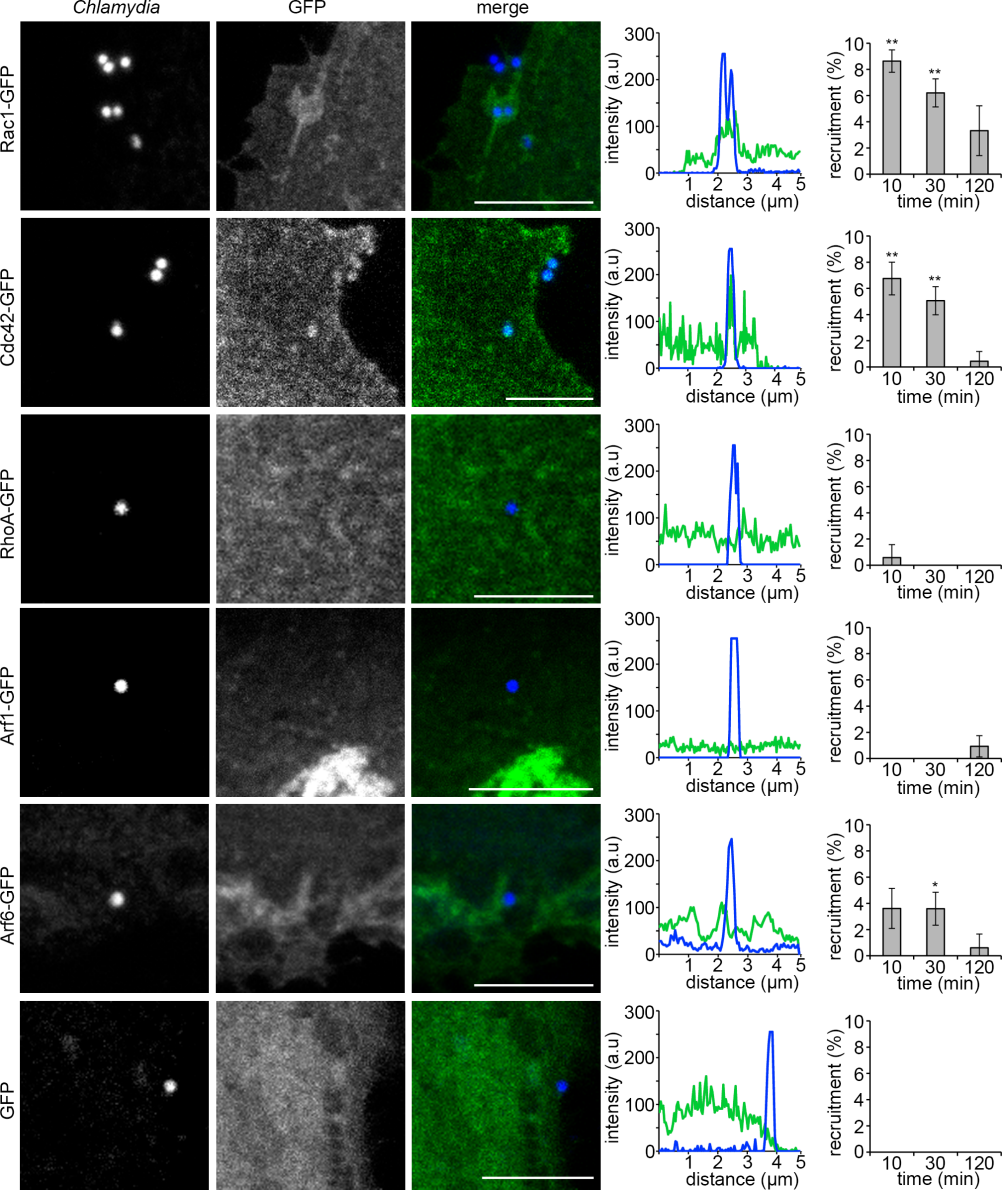
GTPase localisation during *C*. *trachomatis* entry into cultured RPE1 cells. Cultured RPE1 cells were transfected with Rac1-GFP, Cdc42-GFP, RhoA-GFP, Arf1-GFP, Arf6-GFP or GFP alone and infected with *C*. *trachomatis* LGV2 18h later. Cells were infected for up to 120 min prior to fixation. Fixed cells were stained with an anti-*Chlamydia* primary antibody and an Alexa Fluor 633-conjugated secondary antibody and rhodamine phalloidin (red) to visualise F-actin. Scale bars, 5 μm. Representative images show maximum projections of confocal xy sections of the focal plane containing the EB and the nearest neighbour z-sections (±0.2 μm). Line intensity plots taken through the centre of the EB. ≥ 100 cell-associated bacteria were assessed for recruitment of each GFP fusion protein during entry, expressed as the average ±SD (n = 3). P-values obtained from Student's unpaired two-tailed t-test comparing recruitment of GFP at the equivalent timepoint,* P<0.05, ** P<0.01.

To extend the inhibitor screening, it was important to establish whether the implicated signal transducers were specifically recruited to *C*. *trachomatis* entry foci, and to gain insights into the spatio-temporal dynamics of this process. Initially, RPE1 cells transiently expressing Cdc42-GFP, Rac1-GFP, RhoA-GFP, Arf1-GFP or Arf6-GFP were infected with *C*. *trachomatis* LGV2 and observed over a two hour timecourse. In agreement with the inhibitor-based screen, Rac1-GFP and Cdc42-GFP were transiently recruited from 10 minutes post infection (**Fig 3, Rac1-GFP** and **Cdc42-GFP**, and fluorescence intensity plots through the central plane of the EB), whereas Arf1-GFP and RhoA-GFP were not enriched at entry foci (**Fig 3, Arf1-GFP** and **RhoA-GFP**). At this timepoint, only 7–10% of bacteria are intracellular (**S5 Fig**), thus the recruitment of Rac1 and Cdc42 (8.6 ±0.9% and 6.7 ±1.3% respectively) reflects the transient association of these with nearly every invasion-competent EB at 10 minutes post-infection. Arf6-GFP was observed with lower frequency in membrane ruffles adjacent to some EBs (**Fig 3, Arf6-GFP**), consistent with its role in membrane ruffling and Rac1 trafficking [54,55]. No enrichment of endogenous clathrin, caveolin-1 or flotillin-1 was evident at entry foci under equivalent conditions (**S11 Fig, clathrin, caveolin-1** and **flotillin-1**), consistent with the lack of inhibition of inclusion formation by Tip, cholesterol oxidase and filipin (**S6 Fig**). These data support the view that Cdc42 and Rac1 GTPases are dominant early host mediators of *C*. *trachomatis* entry into RPE1 cells.

**Fig 4:**
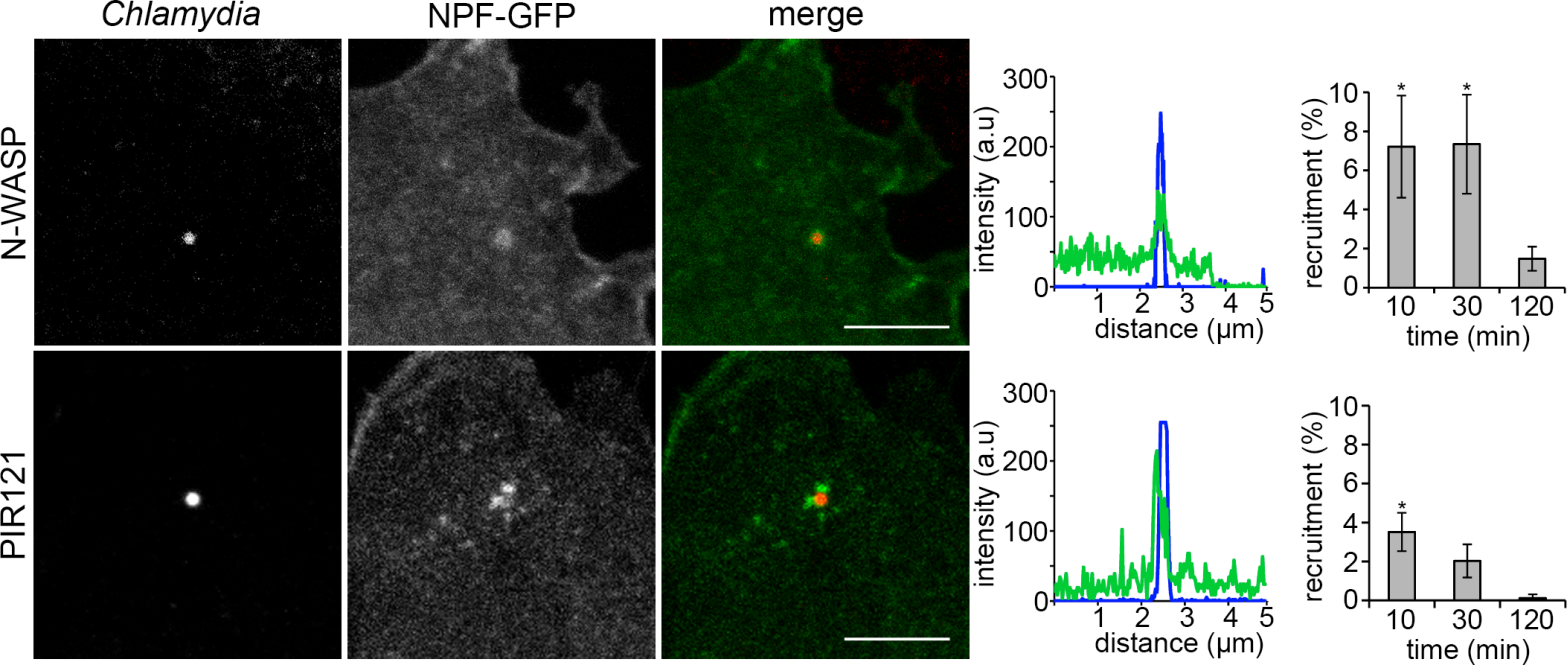
*C*. *trachomatis* associates with NPF-GFP fusion proteins early during infection. RPE1 cells were transfected with constructs expressing GFP-N-WASP or PIR121-GFP (WAVE) and infected with *C*. *trachomatis* LGV2 18 h later. Cells were infected for up to 120 min prior to fixation. Fixed cells were stained with an anti-*Chlamydia* primary antibody and an AlexaFluor 633 conjugated secondary antibody. Representative images show maximum projections of confocal xy sections of the focal plane containing the EB and the nearest neighbour z-sections (±0.2 μm). Scale bars, 5 μm. Line intensity plots taken through the centre of the EB. ≥ 200 cell-associated bacteria were assessed for recruitment of each GFP fusion protein during entry, expressed as the average ±SD (n = 3). P-values obtained from Student's unpaired two-tailed t-test comparing recruitment of GFP at the equivalent timepoint,* P<0.05, ** P<0.01.

Since the NPFs N-WASP and WAVE bridge the activated Rho-family GTPases Cdc42 and Rac1 to the Arp2/3 complex, respectively, we next examined the recruitment of N-WASP and the WAVE complex following *C*. *trachomatis* LGV2 infection of RPE1 cells. The WAVE subunit PIR121-GFP was recruited to entry foci (**Fig 4, PIR121**), consistent with the reported requirement for Rac1 and the WAVE complex during *C*. *trachomatis* infection [38,39]. N-WASP-GFP was recruited more frequently to *C*. *trachomatis* entry foci than PIR121-GFP from as early as 10 minutes post infection (**Fig 4, N-WASP**), in agreement with our findings that N-WASP inhibition can also block bacterial entry (**Fig 2, N-WASP**). There was no apparent specific preference of the GTPase-GFP or NFP-GFP for specific EB-associated F-actin structures.

Given that N-WASP is transiently recruited to *C*. *trachomatis* entry foci and N-WASP inhibition has dose-dependent effects on bacterial entry, we validated the role of N-WASP in entry using knockout mouse embryonic fibroblasts (N-WASP^-/-^). In these cells, chlamydial adhesion was reduced by >70% compared to isogenic wild-type control cells (**S12A Fig**), while *C*. *trachomatis* entry was significantly reduced by 54 ± 17% (**S12B and S12C Fig**). These data confirm the importance of N-WASP during entry processes, yet imply the role of N-WASP is more complex than simply the requirement in entry alone. The fact that upstream adhesion of *C*. *trachomatis* is also affected may be indicative of a role for N-WASP and consequently the actin cytoskeleton in stablising chlamydial adhesion, which may subsequently impact bacterial entry efficiency. Alternatively, the absence of N-WASP in the knockout cells may disturb F-actin organization and influence normal *C*. *trachomatis* adhesion processes indirectly. Nevertheless, these data clearly support the view that N-WASP plays a significant yet previously uncharacterised role in the early interactions between *C*. *trachomatis* and the host cell.

### A macropinocytosis-like pathway underlying *C*. *trachomatis* entry

**Fig 5:**
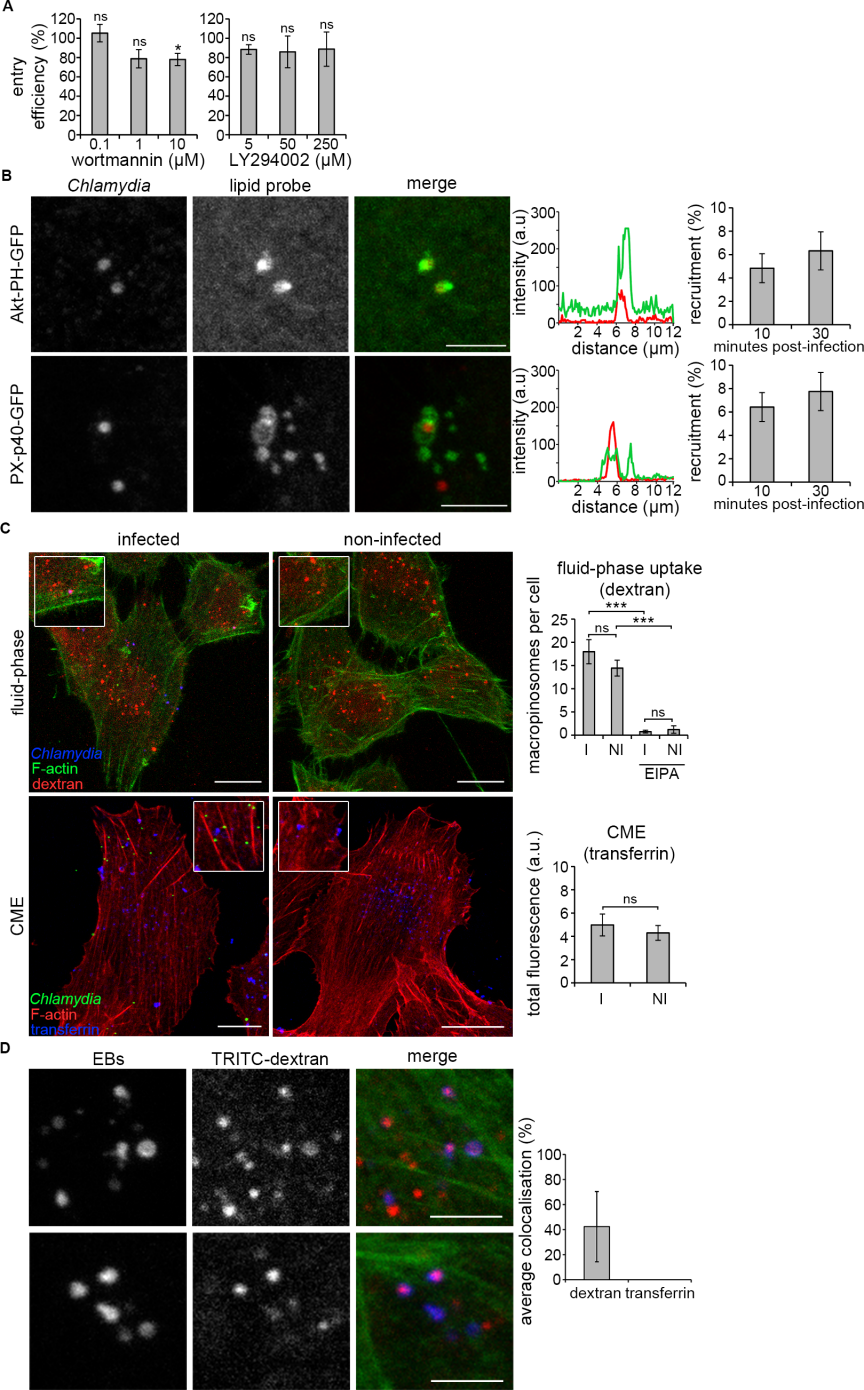
*C*. *trachomatis* EBs are internalised by a macropinocytosis-like pathway. (A) Chlamydial entry is independent of PI3K activity. Cultured RPE1 cells were pretreated with the indicated concentrations of PI3K inhibitors LY294002 or wortmannin for 5 min, followed by infection by *C*. *trachomati*s LGV2 in the presence of inhibitor. At 2 h post-infection the cells were fixed with 1% PFA and stained with an anti-*Chlamydia* primary antibody and an Alexa Fluor 488-conjugated secondary antibody. Cells were then permeablised and stained again using anti-*Chlamydia* primary antibody and an Alexa Fluor 633 conjugated secondary antibody. Intracellular bacteria were labelled with only one fluorophore (AlexaFluor 633). The entry efficiency in the presence of inhibitors relative to mock treated control cells was quantified. ≥ 200 bacteria were assessed at each time point and the percentage entry efficiency of EBs expressed as the average ±SD (n = 3). P-values obtained from Student's unpaired two-tailed t-test, * P<0.05, ‘ns’ not significant. (B) Cultured RPE1 cells were transfected with Akt–PH–GFP [PI(3,4,5)P3] or p40-PX-GFP (PI3P) and were infected with *C*. *trachomatis* LGV2 18 h later for 10 or 30 min prior to fixation. Fixed cells were stained with an anti-*Chlamydia* primary antibody and an Alexa Fluor 546-conjugated secondary antibody (red). Scale bars, 5 μm. Representative images show maximum projections of confocal xy sections of the focal plane containing the EB and the nearest neighbour z-sections (±0.2 μm). The recruitment of phosphoinositide-binding reporters was quantified. ≥ 100 bacteria were assessed at each time point and the percentage recruitment to EBs is expressed as the average ±SD (n = 3). (C) Fluid-phase marker dextran associates with EBs during entry. Representative immunofluorescence images of cultured RPE1 cells incubated for 30 min with TRITC-dextran or transferrin conjugated to Alexa Fluor 647 during infection of cells with *C*. *trachomatis* LGV2 (I) or in non-infected cells (NI) prior to fixation. Fixed cells were stained with an anti-*Chlamydia* primary antibody and an AlexaFluor-conjugated secondary antibody (633 for dextran experiments; 488 for transferrin experiments), and either rhodamine-phalloidin (transferrin experiments) or AlexaFluor 488-conjugated phalloidin (dextran experiments). Scale bars, 10 μm. Fluid phase uptake (dextran) graph showing the number of macropinosomes per cell. Clathrin mediated endocytosis (CME, transferrin) graph showing the amount (in fluorescence arbitrary units) of internalized transferrin obtained from the total fluorescence signal of transferrin conjugated to Alexa Fluor 647 in the z-stacks. ≥ 30 cells were assessed at each time point for fluid-phase uptake and CME, expressed as the average ±SD (n = 3). P-values obtained from Student's unpaired two-tailed t-test,* P<0.05, ** P<0.01, *** P<0.001, ‘ns’ not significant. (D) Representative images of dextran and EB colocalisation in RPE1 cells. Cultured RPE1 cells were infected with *C*. *trachomatis* LGV2 (blue) in the presence of TRITC-dextran (red) for 30 min prior to fixation. Fixed cells were stained with an anti-*Chlamydia* primary antibody and an AlexaFluor 633-conjugated secondary antibody and AlexaFluor 488-conjugated phalloidin. Scale bars, 2.5 μm. Bar chart shows object-based colocalisation analysis of EBs within 5 pixels of transferrin or dextran punctae.

The requirement for Rac1, Cdc42, N-WASP, the Arp2/3 complex and macropinocytosis-associated sodium-proton exchangers inhibited by EIPA, and the dynamic recruitment of Rac1, Cdc42, WAVE and N-WASP to entry foci, together with the association of EBs with phagocytic cups, suggested that *C*. *trachomatis* entry shared many similarities to growth factor and virus-induced macropinocytosis [56]. An additional hallmark of macropinocytosis is the associated activity of phosphoinositol-3-kinase (PI3K), and the sequential generation of the phosphoinositide signalling intermediates phosphatidylinositol-3,4,5-trisphosphate [PI(3,4,5)P3] and phosphatidylinositol-3-phosphate (PI3P) at the plasma membrane. Consequently, we next investigated PI3K activity and used reporters to determine the localisation of 3-phosphoinositide species during chlamydial internalisation. RPE1 cells were infected with *C*. *trachomatis* LGV2 in the presence of the pan-PI3K inhibitors wortmannin and LY294002 [57], and the effect on bacterial entry assessed using the fluorescence ‘inside-out’ assay. Neither wortmannin nor LY294002 significantly inhibited *C*. *trachomatis* entry (**Fig 5A**), in agreement with previous data in HeLa cells showing wortmannin-insensitive chlamydial internalization [2]. However, Akt-PH-GFP, which reports PI(3,4,5)P3 when expressed in cultured cells, was recruited to EBs 10 and 30 minutes post infection (**Fig 5B, Akt-PH-GFP**). Strikingly, the PI3P reporter PX-p40-GFP not only accumulated at entry sites, but also persisted around motile early vacuoles encapsulating EBs (**Fig 5B, PX-p40-GFP** and **S1 Movie**). These data suggest that *C*. *trachomatis* enters via a macropinocytosis-like route, with features subtly distinct from the canonical pathway. This would not be without precedent, as a wortmannin- and LY294002-insensitive pathway generates PI(3,4,5)P3 during *Salmonella* entry [58].

Despite this apparent difference in the requirement for PI3K, 40 ± 26% of *C*. *trachomatis* EBs co-localised with the fluid-phase marker 10,000 MW dextran after 30 minutes, whereas no equivalent association was observed with the CME marker transferrin (**Fig 5C,** and **Fig 5D, compare dextran and transferrin**), consistent with the lack of clathrin recruitment (**S11 Fig, clathrin**). Fluid-phase and transferrin uptake were not significantly enhanced during infection (**Fig 5C,** compare **NI** and **I** panels), although dextran uptake was inhibited by EIPA treatment. Using this approach, it was not possible to determine whether dextran-labelled EB-containing macropinosomes were derived from a defined class of F-actin-rich surface structure. However, when cells were infected with *C*. *trachomatis* in the presence of 70,000 MW dextran, by comparison there did not appear to be equivalent coincidence between 70,000 MW dextran and *C*. *trachomatis* EBs (**S13 Fig**), suggesting a limit in the capacity of the uptake vesicle. This is in accordance with the tight encapsulation of *C*. *trachomatis* EBs observed by cryo-electron tomography at this time point [17]. Taken together, these data reveal that *C*. *trachomatis* LGV2 entry can proceed via atypical macropinocytosis-like events that share hallmarks of the archetypal cellular pathway, yet also exhibit key differences.

### Sorting nexin 9 mediates filopodial capture during *Chlamydia*-induced macropinocytosis

Macropinosomes are derived from membrane ruffles and protrusions folding back and fusing with the plasma membrane to form large vesicles [59]. Although the signalling underlying cellular macropinocytosis is incompletely understood Rac1, WAVE, Cdc42 and N-WASP, along with other downstream effectors such as PAK1, are implicated in this process [56]. Phosphoinositide signalling is also central, with PI(3,4,5)P3 present in phagocytic cups being rapidly dephosphorylated to PI3P on the nascent macropinosome [60,61]. The sorting nexin (SNX) protein family is implicated in membrane trafficking, cargo sorting and endocytosis, and is characterised by a phosphoinositide-binding phox (PX) domain. The PX domain confers phosphoinositide binding specificity, with some PX domains binding PI(3)P [62]. The SNX-PX-BAR subfamily contain a BAR domain, and of these SNX1, SNX5, SNX9, SNX18 and SNX33 are implicated in macropinosome formation [63–65].

To further characterise the macropinocytic-like entry pathway of *C*. *trachomatis*, we explored whether SNX-PX-BAR proteins were involved early during bacterial entry. Cultured RPE1 cells expressing individual SNX-PX-BAR family proteins epitope-tagged at their C-terminus (SNX1-Myc, SNX2-Myc, SNX4-Myc, SNX5-Myc, SNX6-Myc, SNX7-Myc, SNX8-Myc, SNX9-Myc, SNX18-Myc, SNX30-Myc, SNX32-Myc and SNX33-Myc) were infected with C. trachomatis LGV2. Infection was allowed to proceed for 10 minutes prior to fixation and analysis of SNX-PX-BAR-Myc recruitment to entry foci.

**Fig 6:**
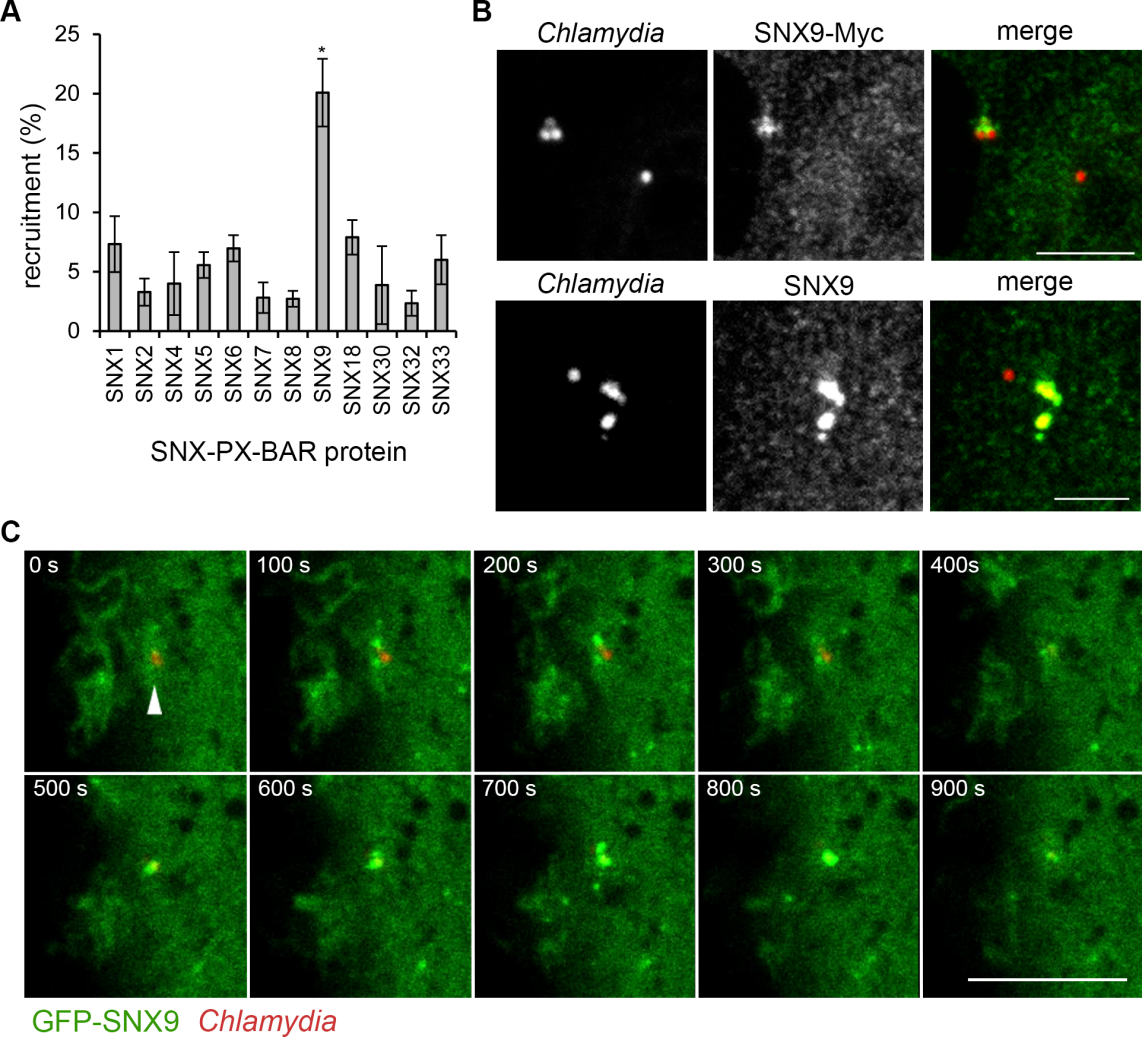
SNX9 is recruited to *C*. *trachomatis* entry sites. (A) Quantification of PX-BAR-Myc recruitment to *C*. *trachomatis* entry sites. Cultured RPE1 cells were transiently transfected with the indicated PX-BAR-Myc fusion proteins and infected with *C*. *trachomatis* LGV2 for 10 min prior to fixation. Fixed cells were stained with an anti-*Chlamydia* primary antibody and an Alexa Fluor 546-conjugated secondary antibody and an anti-Myc primary antibody and an Alexa Fluor 488-conjugated secondary antibody. Recruitment of PX-BAR-Myc proteins was quantified. ≥ 150 cell-associated bacteria were assessed for recruitment of each PX-BAR-Myc fusion protein, expressed as the average percentage ±SD (n = 2). P-values obtained from Student's unpaired two-tailed t-test comparing recruitment of Myc at 10 minutes post-infection,* P<0.05, ** P<0.01. (B) Localisation of PX-BAR-SNX9 and endogenous SNX9 during infection. Cultured RPE1 cells were infected with *C*. *trachomatis* LGV2 for 30 min. Fixed cells were stained with either an anti-Myc or anti-SNX9 primary antibody and an AlexaFluor 488-conjugated secondary antibody. Cells were also stained with an anti-*Chlamydia* primary antibody and an AlexaFluor 546-conjugated secondary antibody. Images are maximum projections of confocal xy sections of the focal plane containing the EB and the nearest neighbour z-sections (±0.2 μm). Scale bars, 2.5 μm. (C) RPE1 cells were transfected with GFP-SNX9 and were infected with *C*. *trachomatis* LGV2 CMTR EBs 18 h later (red). Cells were incubated at 37°C for 15 min prior to imaging. Confocal z-stacks were captured every 10 seconds for 15 min. Panels show maximum projections of confocal z-stacks at 100 s intervals. Scale bar, 7.5 μm.

Of the twelve SNX-PX-BAR-Myc proteins analysed, SNX9-Myc was observed most frequently at entry sites (**Fig 6A, SNX9** and **Fig 6B, SNX9-Myc**). However, since all twelve ectopically-expressed SNX-PX-BAR-Myc derivatives were also present at entry sites at low frequency using our applied scoring criteria (**Fig 6A**), we next verified the localisation of endogenous SNX9 with an anti-SNX9 polyclonal antibody by indirect immunofluorescence. This antibody recognised endogenous SNX9 in accordance with literature (**S14A Fig anti-SNX9**; [66,67]), and within F-actin pedestals generated following infection of RPE1 cells with enteropathogenic *Escherichia coli* (EPEC) (**S14B Fig**; [68]). Endogenous SNX9 was frequently present at sites of EB-host cell interaction in RPE1 cells infected with *C*. *trachomatis* (**Fig 6B, SNX9**), in agreement with the recruitment of SNX9-Myc (**Fig 6B, SNX9-Myc**). In addition to these studies using fixed cells, RPE1 cells expressing GFP-SNX9 were also observed by live imaging. *In trans* expression of GFP-SNX9 expression generated two distinct phenotypes, dependent on the level of expression. High expression induced extensive membrane tubulation, whereas lower expression generated a punctate distribution reminiscent of the endogenous protein (compare left and right GFP-SNX9 panels in **S14A Fig GFP-SNX9**; [66,69]). Consequently, cells exhibiting membrane tubulation were excluded from further analysis. Functionality of GFP-SNX9 in low-expressing cells was additionally confirmed by verifying localisation within F-actin pedestals generated following infection with EPEC (**S14C Fig**). Cultured RPE1 cells expressing low levels of GFP-SNX9 were infected with *C*. *trachomatis* LGV2. GFP-SNX9 was clearly recruited to cell-associated EBs (**Fig 6C**; **S2 Movie**). Live imaging showed that EBs adhere in close proximity to peripheral membrane ruffles enriched in GFP-SNX9, following which intense GFP-SNX9 puncta form that directly overlap with the EB, dissipating ~400 seconds later (**S2 Movie**). These combined approaches reveal the specific and transient recruitment of the SNX-PX-BAR family protein SNX9 to *C*. *trachomatis* entry sites.

### SNX9 is central to filopodial association early during *C*. *trachomatis* entry

**Fig 7:**
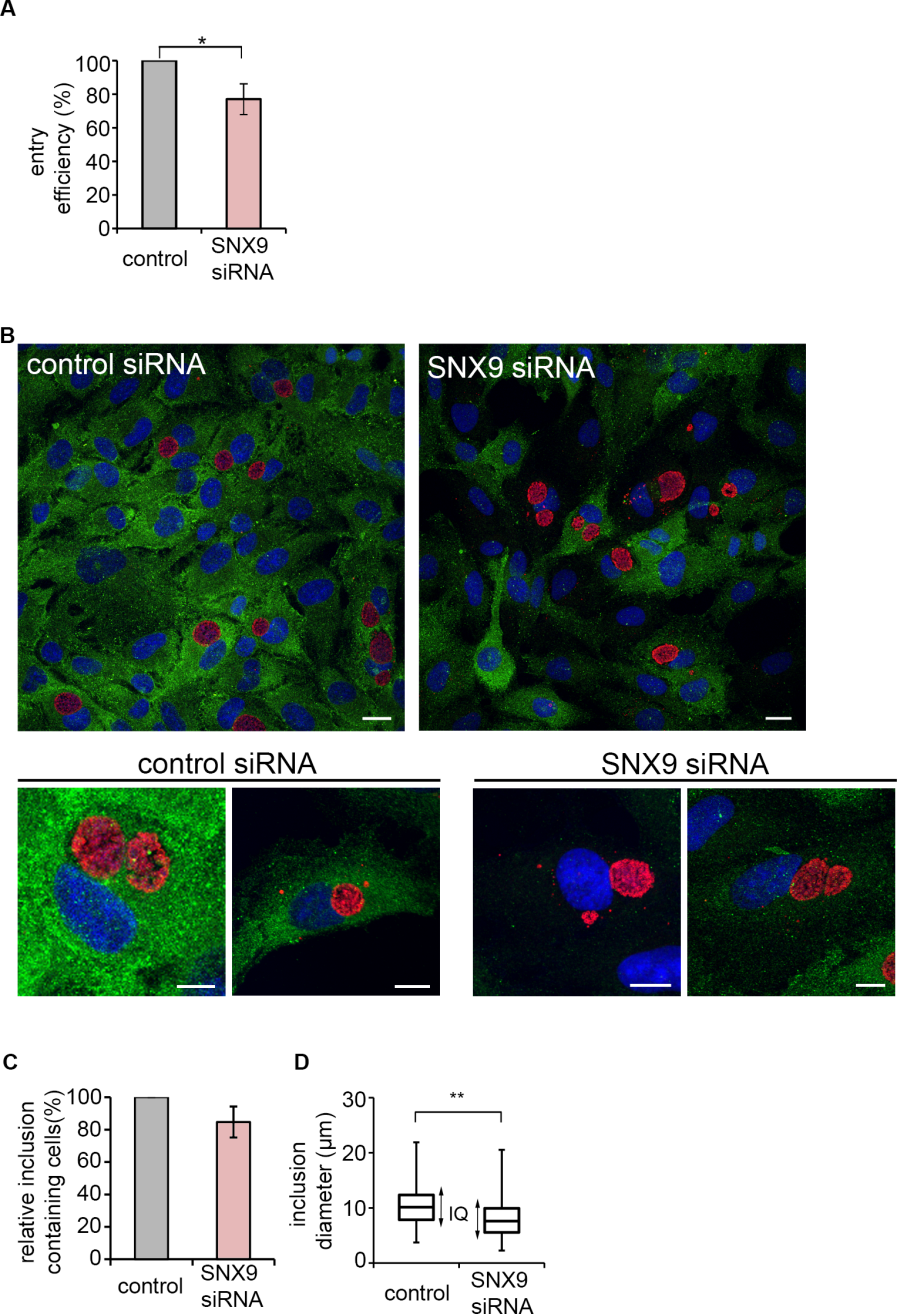
*C*. *trachomatis* infection is attenuated in SNX9 knockdown cells. (A) SNX9 knockdown attenuates bacterial entry into RPE1 cells. Cultured RPE1 cells were treated with siRNA targetting SNX9 or scrambled control siRNA. 72 h later cells were infected with *C*. *trachomati*s LGV2. At 2 h post-infection the cells were washed then fixed with 1% PFA and stained with an anti-*Chlamydia* primary antibody and an Alexa Fluor 488-conjugated secondary antibody. Cells were permeablised and stained again using anti-*Chlamydia* primary antibody and an Alexa Fluor 633-conjugated secondary antibody. Intracellular bacteria were labelled with only one fluorophore (Alexa Fluor 633). Entry efficiency was calculated in SNX9 siRNA treated cells relative to control cells. ≥ 200 cell-associated bacteria were assessed and expressed as the average ±SD (n = 3). P-values obtained from Student's unpaired two-tailed t-test,* P<0.05. (B) Inclusion formation in SNX9 siRNA treated cells. Cultured RPE1 cells were treated with siRNA targetting SNX9 or scrambled control siRNA. 48 h later cells were infected with *C*. *trachomati*s LGV2. At 24 h post-infection the cells were fixed. Cells were stained with anti-SNX9 and anti-*Chlamydia* primary antibodies, followed by Alexa Fluor 488 (green) and 546 (red)-conjugated secondary antibodies respectively and DRAQ5 (blue). Scale bars, 10 μm. (C) Fewer cells are infected in SNX9 knockdown cells. Cells were infected with *C*. *trachomatis* LGV2 for 24 h prior to fixation. Cells were stained with anti-SNX9 and anti-*Chlamydia* primary antibodies, followed by Alexa Fluor 488 (green) and 546 (red)-conjugated secondary antibodies respectively. 10 fields of view SNX9 siRNA or scrambled control siRNA treated cells were analysed for the number of inclusion containing cells. The average percentage of inclusion containing cells in the SNX9 siRNA treated cells relative to scrambled control was calculated ±SD (n = 3). P-values obtained from Student's unpaired two-tailed t-test, * P<0.05. (D) Box and whiskers plot comparing inclusion diameter (μm) in SNX9 siRNA treated cells. Cultured RPE1 cells treated with SNX9 siRNA or scrambled control siRNA were infected with *C*. *trachomatis* LGV2 for 24 h prior to fixation and quantification of inclusion diameter. The whiskers of each plot extend to the minimum and maximum inclusion diameters of the population, whereas the box indicates the interquartile range ‘IQ’ which consists of the middle 50% of the data. Box and whiskers contains pooled data from three independent experiments each analysing ≥ 30 infected cells. P-value obtained from Student's unpaired two-tailed t-test, ** P<0.01.

Given that SNX9 is transiently recruited to *C*. *trachomatis* entry foci, we next investigated the possible roles for SNX9 during infection. The effect of SNX9 knockdown on *C*. *trachomatis* entry was therefore assessed. Cultured RPE1 cells were treated with pooled SNX9 siRNA for 72 hours or equivalently with control non-targeting scrambled siRNA, prior to infection with *C*. *trachomatis* LGV2. Infection was allowed to proceed for two hours prior to fixation and quantification of intracellular bacteria by inside-outside differential fluorescence staining (**Fig 7A**). Knockdown of SNX9 resulted in a 20 ±8% reduction in the number of internalised bacteria (p<0.05) compared to the equivalent non-targeting control siRNA treated population, revealing SNX9 contributes to bacterial entry.

To establish whether the effect of SNX9 knockdown is limited to invasion, cells were treated with siRNA for 48 hours, prior to infection with *C*. *trachomatis* LGV2. Infection was allowed to proceed for 24 hours prior to fixation and quantification of the number of inclusion containing cells (**Fig 7B, Fig 7C**). No significant difference was observed in the overall number of infected cells (**Fig 7C**). However, RPE1 cells treated with SNX9 siRNA had a significant number of smaller-sized inclusions (**Fig 7D**). While the spread of inclusion diameters in control non-targeting siRNA (10.4 ± 3.4 μm) and SNX9 siRNA (8.1 ± 3.8 μm) treated cells were similar, the minimum and maximum inclusion diameters in the SNX9 siRNA treated cells were smaller than those in control non-targeting siRNA treated cells. This suggests that SNX9 influences inclusion growth, or the phenotype could be a secondary effect arising from the reduction in *C*. *trachomatis* entry (**Fig 7A**).

**Fig 8:**
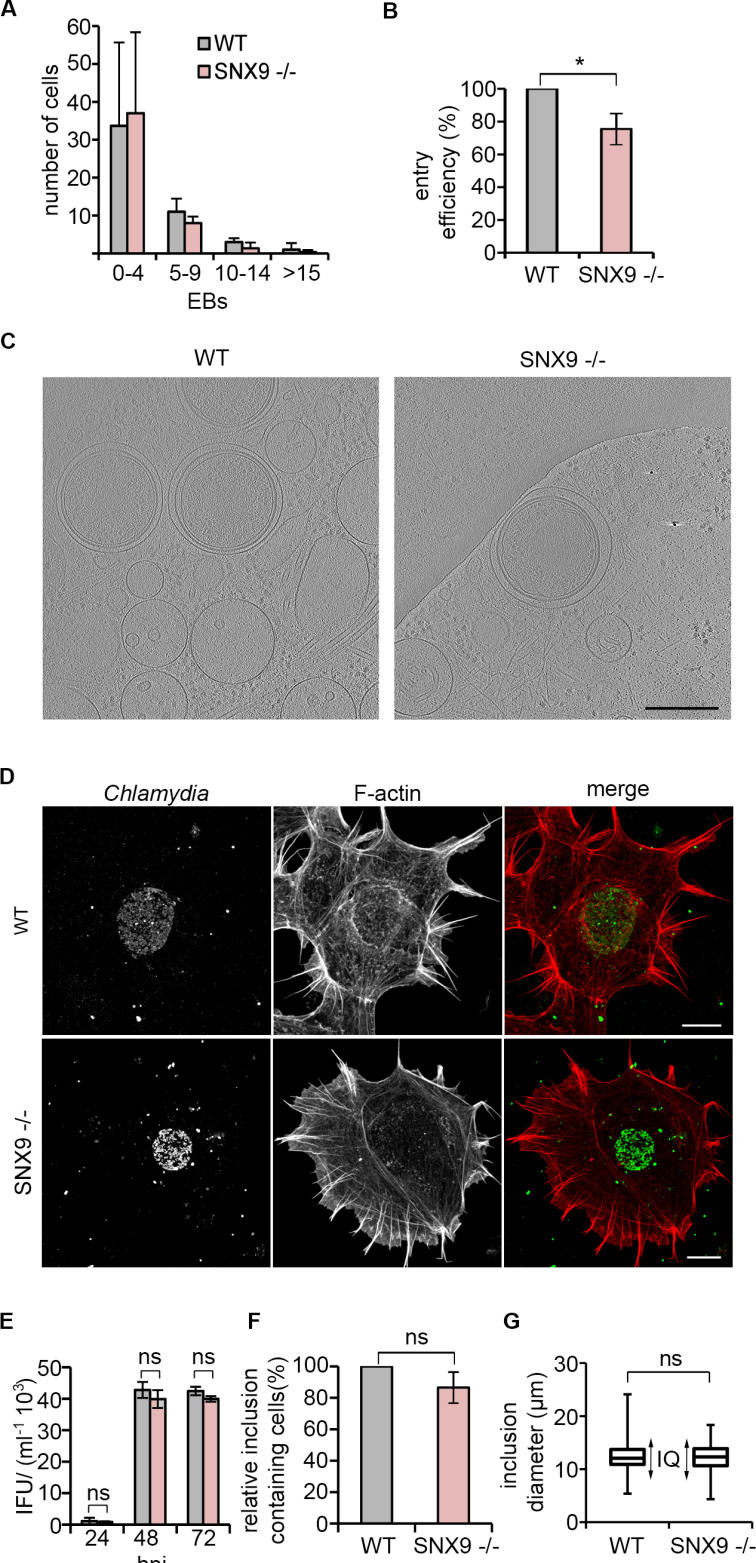
*C*. *trachomatis* entry is attenuated in SNX9 knockout cells but inclusion formation is unaffected. (A) Adhesion is unaffected in SNX9^-/-^ cells. Numbers of adherent bacteria per cell were quantified following an adhesion assay. HAP1 cells were incubated at 4°C for 1 h with infection medium containing *C*. *trachomatis* LGV2 in suspension at an MOI of 100. Cells were washed and fixed. Fixed cells were stained with an anti-*Chlamydia* primary antibody and an AlexaFluor 488-conjugated secondary antibody and the number of adherent bacteria per cell was assessed for 50 cells, expressed as the average ±SD (n = 3). P-value not significant. (B) SNX9 knockout attenuates bacterial entry into HAP1 cells. Cultured HAP1 WT and SNX9 ^-/-^ cells were infected with *C*. *trachomati*s LGV2. At 2 h post-infection the cells were washed then fixed with 1% PFA and stained with an anti-*Chlamydia* primary antibody and an Alexa Fluor 488-conjugated secondary antibody. Cells were permeablised and stained again using anti-*Chlamydia* primary antibody and an Alexa Fluor 633-conjugated secondary antibody. Intracellular bacteria were labelled with only one fluorophore (Alexa Fluor 633). Entry efficiency was calculated in HAP1 SNX9^-/-^ cells relative to HAP1 WT cells. ≥ 200 cell-associated bacteria were assessed and expressed as the average ±SD (n = 3). P-values obtained from Student's unpaired two-tailed t-test,* P<0.05. (C) EB-containing vacuoles form normally in HAP1 SNX9^-/-^ cells. Whole cell cryo-EM tomography was performed as described by Nans et al. (2014) using HAP1 WT and SNX9^-/-^ cells infected with *C*. *trachomatis* LGV2. Cells were infected for 3 h prior to vitrification by plunge-freezing. Images show xy tomographic slices from denoised cryo-electron tomograms of intracellular EBs within early membrane bound-compartments. Scale bars, 300 nm. (D) Inclusion formation is indistinguishable in HAP1 WT and SNX9^-/-^ cells. Cells were infected with *C*. *trachomatis* LGV2 for 24 h prior to fixation. Fixed cells were labelled with an anti-*Chlamydia* primary antibody and an AlexaFluor 488 (green) conjugated secondary antibody and TRITC-phalloidin to visualise F-actin (red). Scale bars, 10 μm. (E) Bacterial replication is unaffected in SNX9^-/-^ cells. Inclusion forming units (IFU) recovered from HAP1 WT or SNX9^-/-^ cells at 24, 48 or 72 h post-infection were used to infect HeLa cells for 24 h and fixed. Fixed cells were labelled with anti-*Chlamydia* primary antibody and an Alexa Fluor 488 conjugated secondary antibody and DRAQ5 (DNA) and the IFU was calculated. P-value obtained from Student's unpaired two-tailed t-test, ‘ns’ not significant. (F) Quantification of number of cells containing inclusions in SNX9^-/-^ cells. Cultured HAP1 WT or SNX9^-/-^ cells were infected with *C*. *trachomatis* LGV2 for 24 h prior to fixation. Fixed cells were stained with an anti-*Chlamydia* primary antibody and an AlexaFluor 488 conjugated secondary antibody and DRAQ5. 10 fields of view (> 400 cells) in HAP1 WT and SNX9^-/-^ cells were analysed for the number of inclusion containing cells. The average percentage of inclusion containing cells in the HAP1 SNX9^-/-^ cells relative to the WT was calculated ±SD (n = 3). P-values obtained from Student's unpaired two-tailed t-test, ‘ns’ not significant. (G) Box and whiskers plot comparing inclusion diameter (μm) in HAP1 WT or SNX9^-/-^ cells. Cultured HAP1 WT or SNX9^-/-^ cells were infected with *C*. *trachomatis* LGV2 for 24 h prior to fixation and quantification of inclusion diameter. The whiskers of each plot extend to the minimum and maximum inclusion diameters of the population, whereas the box indicates the interquartile range ‘IQ’ which consists of the middle 50% of the data. Box and whiskers contains pooled data from three independent experiments each analysing ≥ 50 infected cells. P-value obtained from Student's unpaired two-tailed t-test, ‘ns’ not significant.

However, as these knockdown experiments inevitably represent a mixed population, we exploited human adherent HAP1 cells that recapitulate the chlamydial infection cycle [70] to generate a SNX9 knockout cell line (SNX9^-/-^). While bacterial adhesion remained unchanged in comparison to isogenic controls when SNX9^-/-^ cells were infected *C*. *trachomatis* LGV2 (**Fig 8A**), EB entry was significantly reduced by 25 ± 9% (**Fig 8B**). As the membrane-scission protein dynamin interacts with SNX9 [71,72], we examined the structure of early *C*. *trachomatis*-containing vacuoles at 3 hours post infection of HAP1 wild type and SNX9^-/-^ by cryo-EM tomography [17]. The vacuoles formed in SNX9^-/-^ cells were morphologically indistinguishable from those formed in the wild type background (**Fig 8C**), suggesting SNX9 does not participate in membrane scission or early vacuole formation subsequent to the entry defect. Furthermore, there was no apparent change in appearance or formation of the inclusion (**Fig 8D**), the generation of infectious progeny (**Fig 8E**), the number of infected cells (**Fig 8F**) or the inclusion diameter in the SNX9^-/-^ cells (**Fig 8G**). These phenotypic data using knockout cells thus demonstrate that the functional role of SNX9 is limited to the early phase of EB entry.

**Fig 9:**
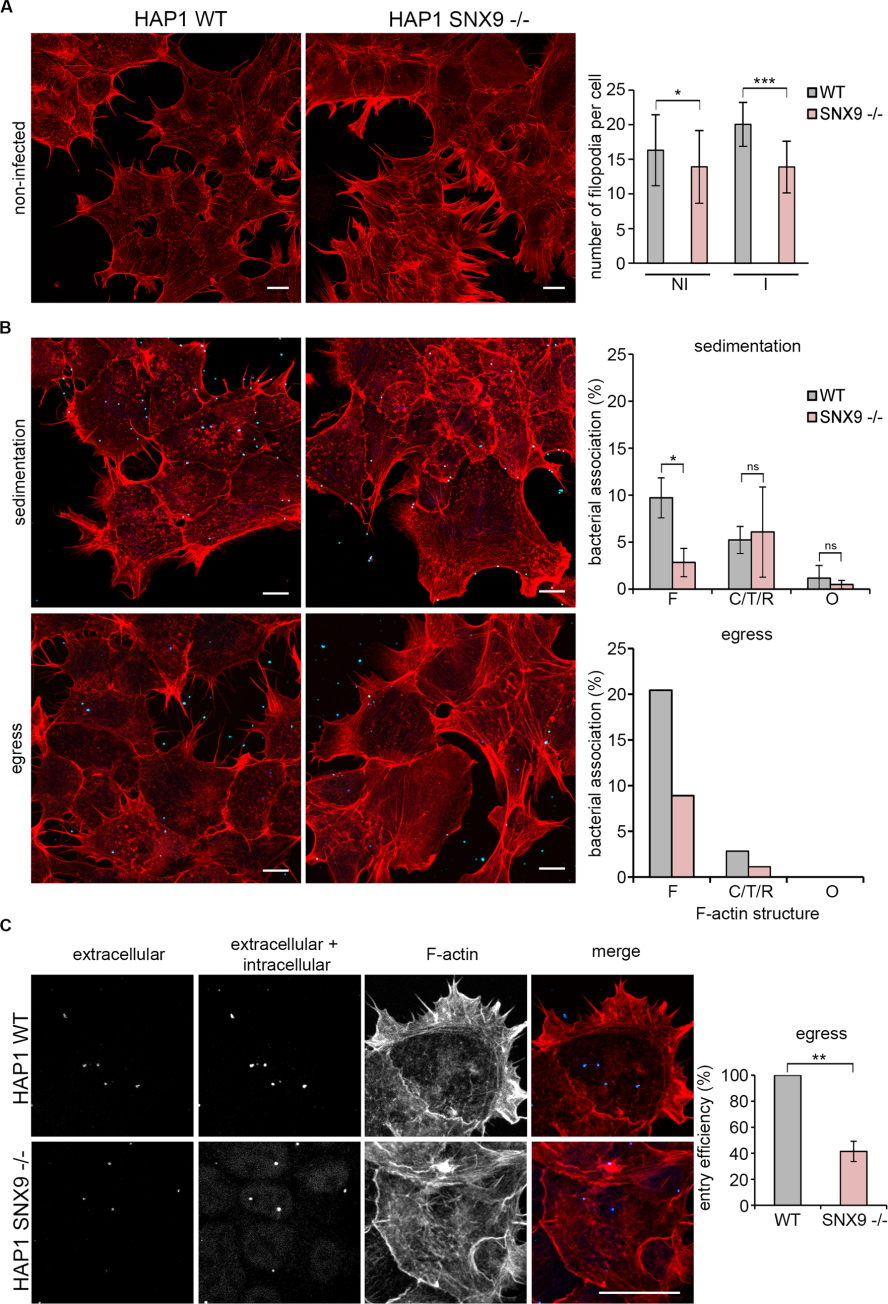
Filopodial capture is significantly attenuated in SNX9^-/-^ cells. (A) Non-infected HAP1 WT and SNX9^-/-^ cells have similar F-actin organisation and filopodia number. Cultured HAP1 WT and SNX9 ^-/-^ cells were fixed and stained with rhodamine-phalloidin to visualise F-actin. Scale bars, 10 μm. The number of filopodia per cell was quantified in maximum projections of confocal xy sections with the criteria that the cells quantified has at least three ‘edges’ not adherent to a neighbouring cell, and that filopodial projections were ≥1 μm in length. 15 cells were assessed for WT and SNX9^-/-^ cells and the results are expressed as the average ±SD (n = 3). P-values obtained from Student's unpaired two-tailed t-test, * P<0.05. (B) The numbers of EBs in association with filopodia during entry is decreased in the HAP1 SNX9^-/-^ cells after infection by sedimentation or egress. Cultured HAP1 WT and SNX9^-/-^ cells were infected with *C*. *trachomati*s LGV2 for 2h. At 2 h post-infection cells fixed with 1% PFA and stained with an anti-*Chlamydia* primary antibody and an Alexa Fluor 488-conjugated secondary antibody. Cells were permeablised and stained again using anti-*Chlamydia* primary antibody and an Alexa Fluor 633-conjugated secondary antibody. Intracellular bacteria were labelled with only one fluorophore (Alexa Fluor 633). Extracellular EBs in association with filopodia, ring, cup/tail, or other F-actin structures were scored, and expressed as the relative percentage of EBs in association with each structure ±SD for ≥150 cell-associated bacteria (n = 3). P-values obtained from Student's unpaired two-tailed t-test,* P<0.05, ‘ns’ not significant. Data from one representative experiment is shown for egress as infection is asynchronous therefore it is inappropriate to cross-compare data from individual experiment for statistical analysis. Scale bars, 10 μm. (C) Entry efficiency is decreased in the SNX9^-/-^ cells. Cultured HAP1 WT and SNX9^-/-^ cells were infected by egress with *C*. *trachomati*s LGV2. At 2 h post-infection the cells fixed with 1% PFA and stained as in ‘(B)’. Representative images are maximum projections of confocal xy sections. Scale bars, 10 μm. Entry efficiency was calculated in HAP1 SNX9^-/-^ cells relative to HAP1 WT cells. ≥ 200 cell-associated bacteria were assessed and expressed as the average ±SD (n = 3). P-values obtained from Student's unpaired two-tailed t-test, ** P<0.01.

SNX9 has an established role in the reorganisation of the actin cytoskeleton, as it interacts directly with multiple proteins that regulate filament dynamics, including the Arp2/3 complex, N-WASP, Cdc42 and RhoA [66,73,74], and is recruited to F-actin-rich structures during fluid-phase endocytosis [73]. To examine whether SNX9 might contribute to cytoskeletal reorganisation during *C*. *trachomatis* entry, F-actin morphology during bacterial entry into wild type and SNX9^-/-^ HAP1 cells was compared using the assays developed previously to assess RPE1 and HeLa cells (**Fig 1** and **S3 Fig**). Adherent wild type and SNX9^-/-^ cells appear phenotypically similar prior to infection, and both populations exhibited protrusions, filopodia and lamellipodia; however, SNX9^-/-^ cells exhibited a small yet significant reduction in the numbers of filopodia per cell in non-infected cells (**Fig 9A**, compare WT and SNX9^-/-^ in ‘NI’ cells). During infection, there was a modest increase in overall filopodia numbers per cell in the WT background, while this was not observed in the SNX9^-/-^ cells, and the reduction in filopodia numbers per cell compared to the WT was greatly reduced (**Fig 9A**, compare WT and SNX9^-/-^ cells during infection ‘I’). This is likely to be an oversimplification however, due to the limitations of confocal microscopy to analyse filopodia on the cell surface, and additional information about the cell surface structures during infection would likely be visible using higher resolution microscopy techniques. When both populations were infected with *C*. *trachomatis* LGV2 and the resulting actin structures compared 30 minutes post infection, the number of bacteria in contact with filopodia decreased by > 50% in the SNX9^-/-^ background, whereas the number of EBs in association with cup, tail, or ring-like structures were not significantly different (**Fig 9B, sedimentation**). As filopodial capture is an early event, we investigated whether this effect occurred only when EBs were artificially sedimented onto the HAP1 cells, by comparing the phenotypes when cells were infected by co-incubation with a population of cells from which bacteria are actively egressing, previously developed for electron tomography [17]. This natural infection captures all the progressive processes that occur during cell entry, rather than experimentally imposing synchronous contact between EBs and the host cell plasma membrane by sedimentation. When wild type and SNX9^-/-^ HAP1 cells were infected in this more natural way, fewer EBs were again observed in contact with filopodia in the SNX9^-/-^ relative to the WT cells (**Fig 9B, egress**). Moreover, entry was strikingly reduced by 59 ± 8% in the SNX9^-/-^ cells (**Fig 9C**), compared to the 25 ± 9% reduction following sedimentation (**Fig 8B**). Taken together, these data demonstrate that SNX9 is required for early filopodia formation during *C*. *trachomatis* entry, an effect that can be partially compensated by artificial sedimentation of the bacteria into contact with the host cells.

## Discussion

In this study, we aimed to further understand the pathways exploited by *C*. *trachomatis* to enter eukaryotic host cells. We demonstrated that during internalisation, distinct F-actin structures interact with cell-associated EBs, including an initial contact between EBs and filopodia, and bacterial association with more complex F-actin cup, tail and ruffle structures. In addition to the recognised roles of the Rac1 GTPase and its associated NPF WAVE, we have revealed an additional unrecognised pathway sharing key hallmarks of macropinocytosis: i) amiloride sensitivity, ii) fluid-phase uptake, iii) recruitment and activity of the NPF N-WASP, iv) the localised generation of phosphoinositide-3-phosphate (PI3P) species and v) involvement of the SNX-PX-BAR protein SNX9 in early filopodial capture.

Macropinocytosis-like pathways underlie cell entry by diverse viruses, including HIV, Vaccinia, Adenovirus, Ebola, Influenza A and the Herpes Simplex Virus [29,75–79]. Indeed, *Chlamydiae* were originally described as a virus due to the absolute requirement of the host cell for survival, yet in many ways *Chlamydiae* remain atypical bacteria, particularly as EBs do not exclusively utilise either zipper or trigger mechanisms of entry [1], and for bacteria they are relatively small. In this study we extend viral parallels to *C*. *trachomatis* entry. We have shown that filopodia associations with EBs occur at a high frequency during invasion in a process reminiscent of the filopodia-mediated capture of viruses, including Vesicular Stomatitis Virus, Murine Leukaemia Virus and HIV [24,80]. In this process, viral particles associate with and either ‘surf’ along or induce filopodial retraction towards the cell body where they are internalised at endocytic ‘hot spots’ [24]. In the context of our data which show a decrease in EB association with filopodia over time, filopodial capture is likely the initial point of contact for EBs and may explain the distinct orientation of the T3SS towards the cell during infection [17], likely facilitating interaction of EBs with the surface of microvilli-dense cells of mucosal membranes they preferentially infect. Indeed, many viruses follow this filopodia-mediated capture with membrane ruffling and uptake via macropinocytosis, furthering similarities between EB and viral uptake. The involvement of a macropinocytosis-like pathway in *C*. *trachomatis* entry is supported by several observations: requirement for Rho GTPases, sensitivity to EIPA, coincidence of EBs and a fluid phase marker during entry, dynamin independence and the association of EBs with PI(3,4,5)P3 and PI3P during early entry [56]. We have also shown a requirement for N-WASP activity during chlamydial entry, suggestive of N-WASP involvement in F-actin rearrangements during internalisation, including filopodia formation [35]. Furthermore, while Cdc42 does not have a major role in *C*. *trachomatis* entry ([38]; this study), there is a limited, although significant reduction in entry upon Cdc42 inhibition. Co-activation of Rac1 and Cdc42 is central to membrane ruffling [81,82] and macropinocytosis [59,83], so the coordinated inter-dependent action of Rac1 and Cdc42 would not be unexpected in membrane ruffling and macropinocytosis-like uptake of *C*. *trachomatis*, especially as a similar pathway underlies *C*. *caviae* entry [84]. Indeed, our data suggest that there was no specific preference of Rac1-, Cdc42, N-WASP or PIR121 (WAVE) GFP reporter fusions for particular EB-associated F-actin superstructures. Further careful investigation of endogenous regulators is now required, as this either reflects a limitation induced by the expression of the reporters or implies functional redundancy.

Further similarities remain to be assessed, such as whether PAK1/2, myosin II, and CtBP1 are involved in entry [85–88], yet *Chlamydia*-specific adaptations would not be unexpected. Typical macropinocytic ruffles are much larger and differences in signalling requirements during macropinocytic uptake of smaller-sized cargoes have been described [87,89,90]. Indeed, despite the clear association of EBs with 3-phosphoinositide species, paradoxically *C*. *trachomatis* entry is largely PI3K-independent. However, this is not without precedent, as for example the association of *Salmonella* with PI(3,4,5)P3-rich membrane ruffles is PI3K-independent, and mediated by as yet unknown mechanisms involving the inositol phosphatase mimic SopB [58].

As expected, cytochalasin D and latrunculin B significantly inhibited inclusion formation and *Salmonella* entry in our hands. However, phalloidin staining revealed F-actin patches accumulated beneath cell-associated EBs, which were reported as internalised by the ‘inside-outside’ assay despite the presence of the inhibitors. Both the F-actin accumulation and this apparent internalisation were unexpected. The latter may merely reflect a difference in antibody accessibility under these conditions, but the accumulation of F-actin may indicate that effector-mediated actin polymerisation might locally limit the effect of actin depolymerising agents. This is not without precedent, as a similar phenomenon was observed after cells infected by *Salmonella typhimurium* were treated with cytochalasin D, where stabilised F-actin patches were similarly evident beneath adherent bacteria [91], and even during bead uptake by phagocytes treated with cytochalasin B [92]. Cytochalasin D may therefore not prevent F-actin accumulation, despite inhibiting actin-dependent processes. As with PI3K- and myosin X-dependent processes, the efficiency of drug-mediated inhibition of actin polymerisation may be dependent on particle size [89,90,93], and consequently the short dense F-actin present beneath ~400 nm diameter EBs represent relatively poor targets. Despite the ‘dogma’ of cytochalasin D-mediated inhibition of pathogen entry, conflicting effects have been reported for *Chlamydia*. Ward and Murray [94] reported only a 50% reduction in chlamydial entry into cytochalasin D-treated cells, whereas Carabeo and colleagues later reported a 41-fold reduction in entry [2]. However, entry of related *C*. *psitacci* was only decreased by 10% [95]. As TARP-mediated actin polymerisation can occur both directly and via Rac1-dependent pathways [8,11], these redundant mechanisms may be differentially susceptible to inhibitors in different strains and target cells. It is clear that the mechanisms by which a small bacterium like *C*. *trachomatis* triggers host actin polymerisation now demands further investigation.

**Fig 10:**
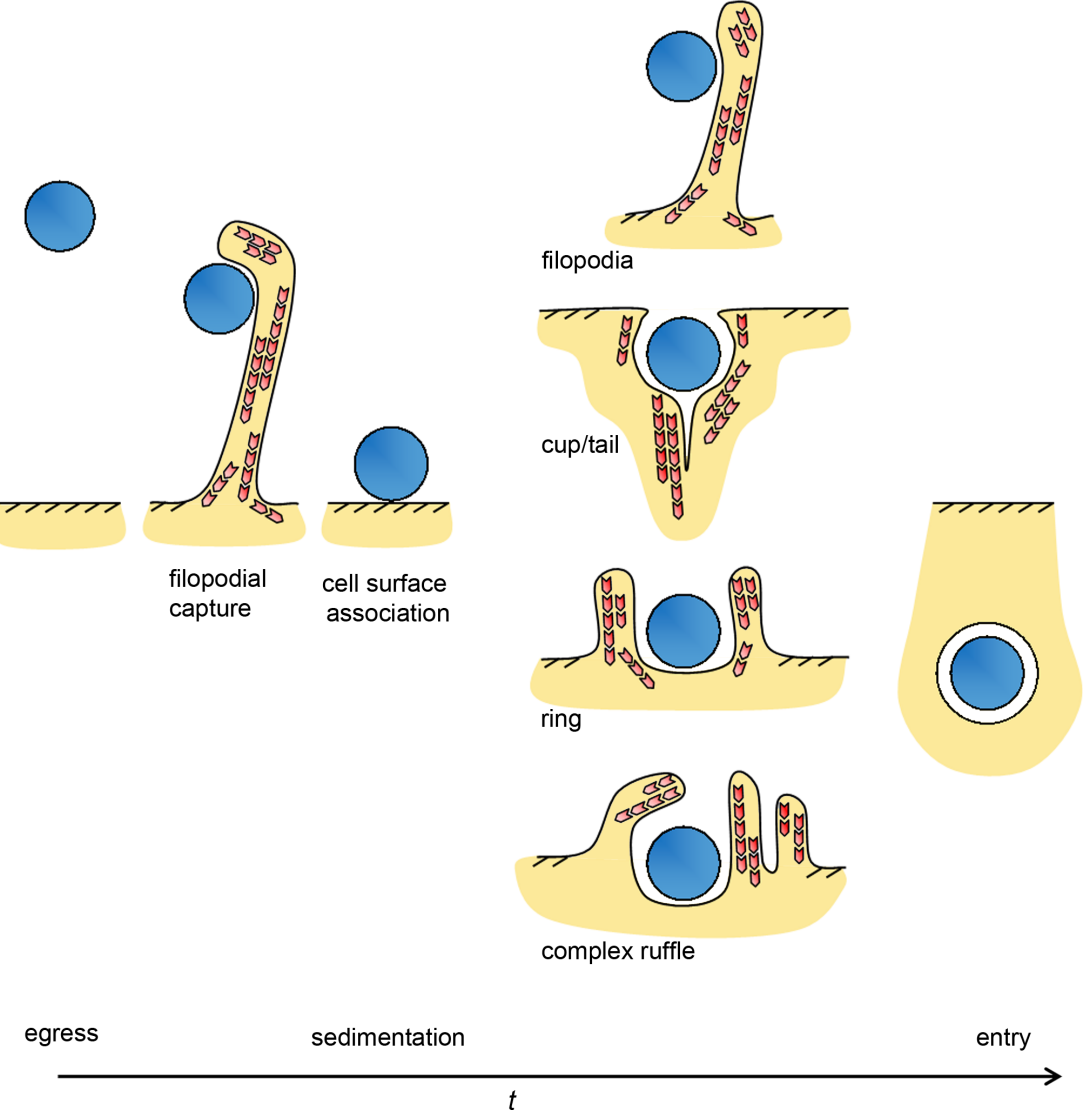
SNX9 knockout decreases *C*. *trachomatis* entry and influences filopodial association early during host F-actin reorganisation. Schematic depicting the entry defects associated with entry into SNX9^-/-^ cells. When bacteria are sedimented onto cells there is a 25 ±9% decrease in *C*. *trachomatis* internalisation, whereas when cells are infected via egress 59 ±8% fewer EBs are internalised. Both methods of infection involve fewer EBs in contact with filopodia. We propose it is this filopodia defect which results in a discrepancy in entry efficiency by the two different methods of infection. This seems likely as filopodial capture would result in more efficient infection when cells are exposed to EBs from infected cells by egress, yet would have less impact on efficiency when cells are infected by sedimentation, consequently partially compensating for SNX9 loss.

We reveal a functional relationship between EB internalisation and PX-BAR-domain containing protein SNX9, which mediates F-actin rearrangements during the early entry process. Indeed, global increases in filopodia formation during infection (**Fig 9A**), similar to those observed previously [2], as well as specific association between *C*. *trachomatis* EBs and filopodia (**Fig 9B**), are decreased in the SNX9^-/-^ cells. How SNX9 is facilitating this process remains an open question, yet one attractive hypothesis is that SNX9 acts as a scaffold for the recruitment and activation of N-WASP to bring about filopodia formation, membrane ruffling and macropinocytic uptake of the EB [66,96,97]. SNX9 interaction with factors important for filopodia formation have been previously identified, and include N-WASP and Arp2/3 [66,73,96]. A more recent study also described a direct interaction between Cdc42 and SNX9 and intriguingly linked SNX9 expression to increased filopodia formation [74], suggesting our observation that initial filopodial capture of EBs is impaired in SNX9^-/-^ cells is a direct consequence of impaired F-actin rearrangements (**Fig 10**).

Similar to the effect of SNX9 depletion on *Salmonella* invasion [58], in our study SNX9 knockdown or knockout did not abrogate entry completely. As some SNX-PX-BAR proteins are reported to have redundant roles, for instance SNX18 can compensate for SNX9 deficiency in CME, which may in part arise from the ability of SNX-PX-BAR family proteins to form heterodimers [98,99], there may be compensatory effects during entry into these SNX9 depleted cells that might also allow chlamydial entry to proceed.

SNX9 is recognised not only for coordination of membrane remodelling, but also as a scaffold to integrate F-actin reorganisation, endocytic traffic and Rho GTPase activity to fulfill roles in both cellular homeostasis and disease [74,100]. Consequently, SNX9 is an attractive candidate for hijack by opportunistic bacterial pathogens. SNX9 has been implicated in both the entry and infection of *Salmonella* and pedestal formation by EPEC and related EHEC [68,69,101,102]. These pathogens utilise T3SS effectors to subvert SNX9 activity, for example during *Salmonella* invasion localised SopB-mediated increases in PI(3,4)P2 recruit SNX9 to membranes to facilitate ruffling and N-WASP signalling. In this process, comparable decreases in *Salmonella* entry are observed when SNX9 is depleted [101]. It is tempting to speculate that a chlamydial effector interacts with SNX9 during cell entry, triggering oligomerisation of SNX9 to amplify SH3-domain mediated interactions akin to the signal amplification induced by EPEC/EHEC [68,69,102]. Whether or not SNX9 is recruited to membranes through an association with a chlamydial T3SS effector remains to be established, but an additional possibility is that manipulation of phosphoinositides during entry temporally and spatially control SNX9 recruitment, similar to the indirect recruitment of SNX9 mediated by *Salmonella* SopB [101]. To date no chlamydial entry effectors that directly interfere with phosphoinositide signalling have been have been identified, unlike in most other bacterial pathogens studied, for example phosphoinositide phosphatase mimics such as *Salmonella* SopB or *Shigella* IpgD [7,103]. However, the T3SS effector TARP binds to the p85 subunit of PI3K, whereas the T3SS effector TepP can interact with both p85 and p110 PI3K subunits and contributes to PI3K activation on early inclusions [11,104]. These effects are intriguing given the apparent PI3K-independent PI3P and PI(3,4,5)P3 interaction with *C*. *trachomatis* we observe during early entry and now warrant further investigation.

Chlamydial entry and early T3SS effectors are not well defined, a fact reinforced by the observation that only *C*. *trachomatis* TARP harbors the N-terminal repeat regions required for Rac1 activation, suggesting additional factors are required by other species [10,16]. In this study we identified roles for N-WASP and macropinocytosis during the internalisation of *C*. *trachomatis* using a panel of small molecule inhibitors, in addition to the established roles of Rac1 and Arp2/3 [38,39]. This is in contrast to other studies which have implicated clathrin mediated endocytosis in EB uptake [105–107]. However, consistent with the data presented here, including a lack of clathrin recruitment to chlamydial entry sites (**S11 Fig**), our detailed dissection of entry structures by cryo-EM never revealed an electron-dense clathrin coat present at chlamydial entry foci [17].

Our data reveal new insights into the diversity of signalling underlying the entry of *C*. *trachomatis* into host cells. We revealed a key initial interaction between EBs and host cell filopodia mediated by the SNX-PX-BAR protein SNX9. This shares similarities with virus-like entry routes, and precedes a macropinocytosis-like pathway. Further studies of the underlying molecular mechanisms will reveal insights into the hijack of host cell function by this important obligate intracellular pathogen.

## Materials and methods

### Reagents

All cell culture reagents, unless otherwise specified, as well as Alexa Fluor dyes and Texas Red-conjugated phalloidin were purchased from Invitrogen. The following primary antibodies were used: mouse anti-chlamydial MOMP-LPS (Argene, 11–114), rabbit anti-*Chlamydia* (Abcam, ab31131), mouse anti-clathrin heavy chain X22 (Thermofischer, MA1-065), rabbit anti-caveolin-1 (BD Biosciences, 610059) mouse anti-flotillin-1 (BD Biosciences, 610821), rabbit anti-myc-tag 71D10 (Cell signalling, mAb #2278) and mouse anti-SNX9 (Abcam, ab118996). Tetramethylrhodamine dextran (TRITC-dextran) 10,000 MW was purchased from Life Technologies and Transferrin Alexa Fluor 647 conjugate was purchased from Thermofischer. Inhibitors used in these experiments were as follows: Rhosin (Rhosin, Calbiochem 555460), EHop (EHop-016, Sigma SML0526), NSC (NSC 23766,Tocris 2161), Secin (SecinH3, Tocris 2849), ML141 (ML 141, Tocris 4266), Casin (Casin, Tocris 3872), EHT (EHT 1864, Tocris 3872), Dynasore (Dynasore, Abcam ab120192), MiTMAB (MiTMAB, Calbiochem 324411), OctMAB (OcTMAB, Tocris 4225), Rho Inhibitor (Rho inhibitor, Cytoskeleton Inc), SMIF (SMIFH2, Tocris 4401), CK636 (CK636, Sigma C7374), CK548 (CK548, Sigma C7499), Lat B (Lantrunculin B, Sigma L5288), Wiskostatin (Wiskostatin, Sigma W2270), Jasplakinolide (Jasplakinolide, Invitrogen J7473), Cyto. D (Cytochalasin D, Sigma C8273), Cholesterol oxidase (cholesterol oxidase, Sigma C5421), Filipin (Filipin, Sigma F4767), Tip (2,4,6-Triiodophenol, Alfa Aesar A17145), EIPA (5-(N-Ethyl-N-isopropyl)amiloride, Sigma A3085).

### Cell culture and transfection

*Homo sapiens* retinal pigment epithelial cells hTERT-RPE-1 (RPE1) cells (ATCC) were cultured in Dulbecco’s Modified Eagle’s Medium/Nutrient Mixture F-12 Ham (DMEM/F12) supplemented with GlutaMax, 10% fetal calf serum (FCS) and penicillin-streptomycin. Cells were transfected with plasmids using Turbofect according to the manufacturer's instructions (Fermentas). Individual plasmids used were SNX1, SNX2, SNX4, SNX5, SNX6, SNX7, SNX8, SNX9, SNX18, SNX30, SNX32, SNX33 (pCi c-Myc), SNX9 (pCi N-EGFP), RhoA (pEGFP-C3), Rac1, Cdc42 (pcDNA3.1-EGFP), Arf1, Arf6, Akt-PH, PLC-PH, SidC, PX-P40 (pEGFP), PIR121 (pDEST), N-WASP (pKC425). Cells were transfected with 20 nM each of SNX9 siRNA, Hs_SNX9_7 SI02777656 and Hs_SNX9_8 SI02777663 (Qiagen) using Hiperfect (Qiagen) according to the manufacturer’s instructions. *Homo sapiens* cervix adenocarcinoma (HeLa), *Cercopithecus aethiops* kidney fibroblast (Cos7) cells (ATCC) and Mouse embryonic fibroblast cells N-WASP^-/-^ (kind gift from Dr Michael Way) were cultured in Dulbecco's modified Eagle's medium (DMEM, high glucose with GlutaMAX) containing 10% FCS and penicillin-streptomycin. *Homo sapiens* adherent fibroblast-like cells derived from male chronic myelogenous leukemia (CML) cell line KBM-7 (HAP1) and HAP1 *SH3PX1*^-/-^ (SNX9^-/-^) cells were purchased from Horizon Genomics. HAP1 cells were cultured in Iscove's Modified Dulbecco's Medium (IMDM) supplemented with 10% FCS and penicillin/streptomycin.

### Infection of cultured cells with *Chlamydia*

*C*. *trachomatis* LGV2 or *C*. *trachomatis* D serovars were propagated in HeLa cells as previously described and stored in SPG buffer at −80°C [108]. Infections were carried out by diluting the stored LGV2 serovar in infection medium (DMEM/F12 or DMEM, 10% FCS, 25μg ml^−1^ gentamicin) resulting in a multiplicity of infection (MOI) of ~5–30 EBs per cell. In sedimentation infection cells seeded 24 h previously were overlaid with infection medium and centrifuged at 900 x *g* for 10 min. At an appropriate time-point, cells were fixed with paraformaldehyde (PFA) for immunostaining. Bacterial inclusion-forming units (IFU) were determined as described [109]. For the egress method, cells were infected according to [17] with minor modifications. Briefly, RPE1 or HAP1 cells were seeded into 100 mm dishes. 24 h later cells were infected with *C*. *trachomatis* LGV2 (MOI 5–30) by sedimentation. The following day, non-infected cells (RPE1 or HAP1) were seeded onto 12 mm coverslips in a separate 24-well plate. At 48 hpi when infected cells begin to release new EB progeny, the coverslips were introduced to the 100 mm dish and incubated for an appropriate time-point at 37°C to allow infection directly with the released EBs.

### Infection of cultured cells with enteropathogenic *E*. *coli*

Enteropathogenic *E*. *coli* (EPEC) was inoculated into Luria-Bertani broth and incubated overnight at 37°C with shaking. EPEC overnight cultures were diluted 1:25 into DMEM supplemented with 10% FCS and 100 mM HEPES pH 7.4 and grown to mid-log phase, conditions which maximise T3SS activity [110]. Cell monolayers were infected with an appropriate dilution of mid-log phase cultures in DMEM supplemented with 10% FCS and 25 mM HEPES pH 7.4 without antibiotics at an MOI of 50. Infected cells were incubated for 4 h at 37°C prior to fixation.

### Infection of cultured cells with *Salmonella enterica*

*Salmonella enterica* overnight cultures were diluted 1:25 in 2 ml of Luria-Bertani broth and incubated for 4 h at 37°C with shaking. Cell monolayers were pre-treated with media without antibiotics containing 1% (v/v) serum and 2μm cytochalasin D. After 5 minutes, this media was replaced with medium containing appropriately diluted bacterial stock and cytochalasin D at an MOI of 50. Cells were centrifuged at 160 × *g*, 10 minutes, room temperature to synchronize the infection. After incubation at 37°C 5% CO2 for 1 h, cells were washed three times and remaining extracellular bacteria killed by incubation of the cells in infection media supplemented with 25 μg/ml gentamicin 37°C 5% CO2, for 1 h. Cells were washed again and lysed in 0.05% (v/v) Triton-X-100. Serial dilutions of cell lysates were plated on LB agar and the percentage of intracellular bacteria compared with the original inoculum was determined for both cytochalasin D treated and mock treated cells.

### Immunofluorescence and live-cell microscopy

For immunolabelling, cells were cultured on 12 mm coverslips in 24-well dishes. When appropriate, cells were fixed by exchanging media for 4% paraformaldehyde (PFA) in phosphate buffered saline (PBS) at room temperature. Cells were incubated in PFA for 20 min before neutralisation using an equal volume of 50 mM NH_4_Cl in PBS for at least 20 min prior to antibody labelling. Following fixation, cells were permeabilized in 0.05% Triton X-100 (10 min), rinsed in PBS and then washed in PBS containing 0.1% (w/v) BSA. Primary antibodies were diluted in PBS / 1% (w/v) BSA, added to coverslips and incubated for 2 h. Secondary antibodies (Alexa Fluor 488, 546 and 633) or Texas Red phalloidin (for visualisation of F-actin) were diluted in PBS / 1% (w/v) BSA, added to coverslips and incubated for 1 h. Coverslips were mounted with Mowiol (Sigma) and observed using a confocal microscope (TCS Sp5 AOBS; Leica). For live-cell imaging of GFP-SNX9 expressing cells, cells were infected as described above with CellTracker CMTR (Thermofischer) labelled *C*. *trachomatis* LGV2 EBs, according to the method devised by Boleti and colleagues [111].

### Invasion efficiency assay

At an appropriate time-point, infected cells were fixed in 1% PFA for 20 min and quenched with 50 mM NH_4_Cl in PBS. Following fixation, cells were rinsed in PBS and then in PBS containing 0.1% (w/v) BSA. Cells were stained as above, incubating with anti-*Chlamydia* primary antibody, followed by secondary antibody (Alexa Fluor 488). Cells were then permeablised in 0.05% Triton X-100 for 10 min. Cells were then stained again with the same anti-*Chlamydia* primary antibody followed by secondary antibody (Alexa Fluor 633, pseudocoloured blue) and Texas-Red phalloidin. Extracellular bacteria are therefore labelled twice with Alexa Fluor 488 (green) and 633 (blue) appearing cyan and allowing them to be distinguished from intracellular bacteria labelled only with Alexa Fluor 633 which appear blue. The percentage of intracellular bacteria was then calculated [total bacteria–extracellular bacteria]/total bacteria x 100) and expressed relative to control.

### Small molecule inhibitor assays

RPE1 cells were plated into 24-well plates containing glass coverslips. 24 h later the cell media was removed and replaced with media containing 1% (v/v) serum and inhibitors at the indicated concentrations. After 5 min, this media was replaced with media containing *C*. *trachomatis* EBs and the inhibitor. Cells were infected as described above and incubated at 37°C for 2 h. For entry experiments at 2 h post-infection, quantification of invasion efficiency was carried out as described previously in inhibitor and mock-treated control cells. For quantification of the effects on inclusion formation, inhibitor was washed out at 2 h post-infection, and the infection allowed to proceed until 24 h post-infection when cells were fixed and stained.

### Dextran and transferrin uptake assays

Cells were seeded at an appropriate density in 24-well plates containing 12 mm coverslips. 24 h later cells were infected as described above in infection media containing 1% serum and either TRITC-dextran at a concentration of 1 mg ml^-1^ for 30 min or transferrin Alexa Fluor 647 conjugate at a concentration of 200 μg ml^-1^ for 30 min. Following ligand uptake, the plates were incubated on ice and the medium was removed. Cells were then washed twice with ice-cold PBS and for 2 min in ice-cold stripping buffer containing 150 mM NaCl, 100 mM glycine, 5 mM KCl and 1 mM CaCl_2_ at pH 4.5. Cells were washed twice with ice-cold PBS prior to fixation in PFA. The total fluorescence of transferrin Alexa Fluor 647 conjugate within cell boundaries was corrected by background fluorescence in ImageJ. Mean transferrin fluorescence in arbitrary units was quantified in infected and non-infected cells. Dextran uptake was measured according to a modified version of the protocol reported by Wang and colleagues [112]. Briefly, the 3D objects counter tool in ImageJ was used to quantify dextran uptake as 3D objects or puncta (macropinosomes) within z-stacks, using a minimum size filter of 0.2 μm2–20 μm^2^ for specificity of macropinosome identification and to minimise background fluorescence.

## Supporting information

S1 Fig Schematic representation of the stages for differential labelling of EBs for the invasion efficiency assay. 1. Cultured cells were infected with *C*. *trachomatis* prior to fixation with 1% paraformaldehyde. 2. Fixed cells were stained with an anti-*Chlamydia* primary antibody and an Alexa Fluor 488-conjugated secondary antibody. 3. Cells were permeabilised with 0.05% Triton X-100 (v/v). 4. The bacteria were stained again using the same anti-*Chlamydia* primary antibody and an Alexa Fluor 633-conjugated secondary antibody. Intracellular bacteria were labelled with only Alexa Fluor 633 (dark blue) extracellular bacteria were labelled with Alexa Fluor 488 and Alexa Fluor 633 (green + blue, cyan; ‘extracellular’ and ‘intracellular and extracellular’ panels). Lower panels indicate an example of the resulting immunofluorescence images stained using this methodology. F-actin was stained with rhodamine-phalloidin.
(TIF)

S2 Fig Additional examples of F-actin classification in cultured RPE1 cells. Left hand panels show diagrammatic representations of filopodia, ring and cup/tails corresponding to the F-actin structures visualised by fluorescence microscopy of cultured cells infected with *C*. *trachomatis*. Representative immunofluorescence images show cultured RPE1 cells infected with *C*. *trachomatis* LGV2 for 30 min prior to fixation. Fixed cells were stained with an anti-*Chlamydia* primary antibody and an Alexa Fluor 488-conjugated secondary antibody and rhodamine phalloidin.
(TIF)

S3 Fig F-actin recruitment to *C*. *trachomatis* entry sites in cultured HeLa cells. (A) Representative immunofluorescence images of F-actin recruitment to *C*. *trachomatis* EBs during early interaction with HeLa cells. Cultured HeLa cells were infected with *C*. *trachomatis* LGV2 for 30 minutes prior to fixation with 1% paraformaldehyde. Fixed cells were stained with an anti-*Chlamydia* primary antibody and an Alexa Fluor 488-conjugated secondary antibody. Cells were permeabilised with 0.05% Triton X-100 (v/v) and the bacteria stained using the same anti-*Chlamydia* primary antibody and an Alexa Fluor 633-conjugated secondary antibody. Intracellular bacteria were labelled with only Alexa Fluor 633 (dark blue; ‘intracellular and extracellular’ panel), extracellular bacteria were labelled with Alexa Fluor 488 and Alexa Fluor 633 (green + blue, cyan; ‘extracellular’ and ‘intracellular and extracellular’ panels). F-actin was stained with rhodamine-phalloidin. White arrowheads show typical examples of indicated classes of F-actin structure. Images are maximum projections of confocal xy sections. Scale bars, 5 μm. Right hand panels show diagrammatic representations of the defined classes of F-actin structures visualised by fluorescence microscopy of cultured HeLa cells infected with *C*. *trachomatis*. (B) Quantification of F-actin structures associated with extracellular *C*. *trachomatis* EBs from 10–120 min post-infection of HeLa cells. Cultured HeLa cells were infected with C. *trachomatis* LGV2 for 10, 30, and 120 min prior to fixation with 1% paraformaldehyde. Fixed cells were stained as above and the association of EBs with the defined F-actin classes was quantified. ≥ 200 bacteria were assessed at each time point and the percentage of EBs in association with each class of structure was calculated, expressed as the average ±SD (n = 3). ≥ 200 bacteria were assessed at each time point and the percentage of EBs in association with each class of structure was calculated, expressed as the average ±SD (n = 3). * P<0.05, ** P<0.01, ‘ns’ not significant using one-way ANOVA followed by a Tukey's post hoc test.
(TIF)

S4 Fig F-actin recruitment to *C*. *trachomatis* serovar D entry sites in cultured RPE1 cells. (A) Representative immunofluorescence images of F-actin recruitment to *C*. *trachomatis* serovar D EBs during early interaction with RPE1 cells. Cultured RPE1 cells were infected with *C*. *trachomatis* LGV2 prior to fixation with 1% paraformaldehyde. Fixed cells were stained with an anti-*Chlamydia* primary antibody and an Alexa Fluor 488-conjugated secondary antibody. Cells were permeabilised with 0.05% Triton X-100 (v/v) and the bacteria stained using the same anti-*Chlamydia* primary antibody and an Alexa Fluor 633-conjugated secondary antibody. Intracellular bacteria were labelled with only Alexa Fluor 633 (dark blue; ‘intracellular and extracellular’ panel), extracellular bacteria were labelled with Alexa Fluor 488 and Alexa Fluor 633 (green + blue, cyan; ‘extracellular’ and ‘intracellular and extracellular’ panels). F-actin was stained with rhodamine-phalloidin. White arrowheads show typical examples of indicated classes of F-actin structure. Images are maximum projections of confocal xy sections. Scale bars, 5 μm. Right hand panels show diagrammatic representations of the defined classes of F-actin structures visualised by fluorescence microscopy of cultured RPE1 cells infected with *C*. *trachomatis* serovar D. (B) Quantification of F-actin structures associated with extracellular *C*. *trachomatis* EBs at 30 min post-infection of RPE1 cells. Cultured RPE1 cells were infected with C. *trachomatis* serovar D for 30 min prior to fixation with 1% PFA. Fixed cells were stained as above and the association of EBs with the defined F-actin classes was quantified. ≥ 200 bacteria were assessed and the percentage of EBs in association with each class of structure was calculated, expressed as the average ±SD (n = 3).
(TIF)

S5 Fig The kinetics of *C*. *trachomatis* LGV2 entry into cultured RPE1 and HeLa cells. (A) Cultured RPE1 cells were infected with *C*. *trachomatis* LGV2 for the indicated timepoints prior to fixation with 1% paraformaldehyde. Fixed cells were stained with an anti-*Chlamydia* primary antibody and an Alexa Fluor 488-conjugated secondary antibody. Cells were permeabilised with 0.5% Triton X-100 (v/v) and the bacteria stained using the same anti-*Chlamydia* primary antibody and an Alexa Fluor 633-conjugated secondary antibody. Intracellular bacteria were labelled with only Alexa Fluor 633, extracellular bacteria were labelled with Alexa Fluor 488 and Alexa Fluor 633. The numbers of intracellular bacteria at each time point was quantified. ≥ 300 bacteria were assessed at each time point and the percentage of intracellular bacteria is expressed as the average ±SD (n = 3). Right hand pie charts indicate the average percentage of F-actin associated extracellular EBs at the indicated timepoint. (B) Cultured HeLa cells were infected with *C*. *trachomatis* LGV2 for the indicated timepoints prior to fixation with 1% paraformaldehyde. Fixed cells were stained with an anti-*Chlamydia* primary antibody and an Alexa Fluor 488-conjugated secondary antibody. Cells were permeabilised with 0.5% Triton X-100 (v/v) and the bacteria stained using the same anti-*Chlamydia* primary antibody and an Alexa Fluor 633-conjugated secondary antibody. Intracellular bacteria were labelled with only Alexa Fluor 633, extracellular bacteria were labelled with Alexa Fluor 488 and Alexa Fluor 633. The numbers of intracellular bacteria at each time point was quantified. ≥ 300 bacteria were assessed at each time point and the percentage of intracellular bacteria is expressed as the average ±SD (n = 3). Right hand pie charts indicate the average percentage of F-actin associated extracellular EBs at the indicated timepoint.
(TIF)

S6 Fig The influence of small-molecule inhibitors on inclusion formation when present during the first 2 h post-infection. Cultured RPE1 cells were pre-treated for 5 min with the indicated concentration of the inhibitor, followed by infection with *Chlamydia trachomati*s LGV2 in the presence of inhibitor. At 2 h post-infection the inhibitor was washed out and infection was allowed to progress for a further 22 h (*i*.*e*. until 24 h post-infection). Cells were then fixed and stained with an anti-*Chlamydia* primary antibody and an Alexa Fluor 488-conjugated secondary antibody. The average number of inclusion-containing cells was quantified for 10 fields of view in both inhibitor and mock treated samples at the three indicated concentrations (μM), expressed as the relative percentage of inclusion containing cells ±SEM (n = 2). A dotted line is drawn at ≥ 75%, indicating where inhibitors decreased inclusion formation by ≥ 25% of control cells. P-values obtained from Student's unpaired two-tailed t-test, * P<0.05, ** P<0.01, *** P<0.001, ‘ns’ not significant.
(TIF)

S7 Fig The influence of small-molecule inhibitors on inclusion morphology when present during the first 2 h post-infection. Cultured RPE1 cells were pre-treated for 5 min with the mid concentration of the indicated inhibitor, followed by infection with *Chlamydia trachomati*s LGV2 in the presence of inhibitor (or equivalent mock control treatments). At 2 h post-infection the inhibitor was washed out and infection was allowed to progress for a further 22 h (*i*.*e*. until 24 h post-infection). Cells were then fixed and stained with an anti-*Chlamydia* primary antibody and an Alexa Fluor 488-conjugated secondary antibody (green) and rhodamine-phalloidin (red). Images are maximum projections of confocal xy sections. Scale bars, 10 μm.
(TIF)

S8 Fig Cytotoxicity in the presence of small molecule inhibitors. (A) and (B) Cultured RPE1 cells were treated for 2 h with either the mid or high concentration of the indicated inhibitor (see **S3 Fig**). Viable cells (non-permeable to trypan blue) were enumerated for inhibitor and mock-treated cells (n = 2) and the percentage viable cells relative to mock-treated control cells was calculated.
(TIF)

S9 Fig *C*. *trachomatis* invasion in the presence of small molecule inhibitors. Cultured RPE1 cells were pre-treated for 5 min with the indicated concentration of the inhibitor, followed by infection with *Chlamydia trachomati*s LGV2 in the presence of inhibitor. At 2 h post-infection the cells were washed then fixed with 1% PFA and stained with an anti-*Chlamydia* primary antibody and an Alexa Fluor 488-conjugated secondary antibody (green). Cells were permeabilised with 0.05% Triton X-100 (v/v) and the bacteria stained using the same anti-*Chlamydia* primary antibody and an Alexa Fluor 633-conjugated secondary antibody. Intracellular bacteria were labelled with only Alexa Fluor 633 (dark blue; ‘intracellular and extracellular’ panel), extracellular bacteria were labelled with Alexa Fluor 488 and Alexa Fluor 633 (green + blue, cyan; ‘extracellular’ and ‘intracellular and extracellular’ panels). Rhodamine phalloidin (red) visualises F-actin. Images are maximum projections of confocal xy sections. Scale bars, 10 μm. The entry efficiency in the presence of inhibitors relative to mock treated control cells was quantified. ≥ 300 bacteria were assessed at each time point and the percentage entry efficiency of EBs is expressed as the average ±SD (n = 3). P-values obtained from Student's unpaired two-tailed t-test, * P<0.05, ** P<0.01, ‘ns’ not significant.
(TIF)

S10 Fig The influence of cytochalasin D on *Salmonella enterica* entry and F-actin distribution. (A) RPE1 cells were pre-treated for 5 minutes with 2 μM cytochalasin D and infected with *Salmonella enterica* serovar typhimurium SL1344 in infection medium containing cytochalasin D. Infection was allowed to proceed for 1 h before cells were washed and further incubated for 1 h in gentamicin-containing cell culture medium. Cells were then lysed and serial dilutions of the resulting lysate were plated onto solid agar. The number of colonies recovered from cytochalasin D and mock-treated control cells was quantified. Results are expressed as a percentage relative to mock treated control ±SD (n = 3). (B) Non-infected RPE1 cells treated with cytochalasin D for 1 h at the indicated concentration. Cells were then fixed and stained with rhodamine-phalloidin to visualize F-actin.
(TIF)

S11 Fig Localisation of endogenous endocytic pathway markers in relation to *C*. *trachomatis* during early infection. Cultured RPE1 cells were infected with *C*. *trachomatis* LGV2 for 10, 30 or 120 min prior to fixation. Fixed cells were stained with an anti-*Chlamydia* primary antibody and an Alexa Fluor 546-conjugated secondary antibody (red) and primary antibodies corresponding to clathrin, caveolin-1 or flotillin-1 followed by an Alexa Fluor 488-conjugated secondary antibody (green). Representative images are from 30 min post-infection and show the whole cell with a close-up of an EB-containing region and are maximum projections of confocal xy sections of the focal plane containing the EB and the nearest neighbour z-sections (±0.2 μm). Scale bars, 10 μm. ≥ 100 cell-associated bacteria were assessed for recruitment of the markers during entry, expressed as the average ±SD (n = 3).
(TIF)

S12 Fig *C*. *trachomatis* adhesion and entry is attenuated in N-WASP knockout cells. **(A)** Adhesion is greatly attenuated in N-WASP^-/-^ mouse embryonic fibroblasts. Numbers of adherent bacteria per cell were quantified following an adhesion assay. N-WASP knockout MEFs were incubated at 4°C for 1 h with infection medium containing *C*. *trachomatis* LGV2 in suspension at an MOI of 100. Cells were washed and fixed. Fixed cells were stained with an anti-*Chlamydia* primary antibody and an AlexaFluor 488-conjugated secondary antibody and the number of adherent bacteria per cell was assessed for ≥ 25 cells, expressed as the average ±SD (n = 3). P-values obtained from Student's unpaired two-tailed t-test, *** P<0.001. (B) Entry efficiency is decreased in N-WASP knockout cells. Immunofluorescence examples of cultured WT and N-WASP^-/-^ mouse embryonic fibroblasts infected with *C*. *trachomati*s LGV2. At 2 h post-infection the cells were washed then fixed with 1% PFA and stained with an anti-*Chlamydia* primary antibody and an Alexa Fluor 488-conjugated secondary antibody. Cells were permeablised and stained again using anti-*Chlamydia* primary antibody and an Alexa Fluor 633-conjugated secondary antibody. Intracellular bacteria were labelled with only one fluorophore (Alexa Fluor 633). Scale bars, 10 μm. (C) Cells were infected and stained as above. Entry efficiency was calculated in N-WASP^-/-^ cells relative to WT cells. ≥ 200 cell-associated bacteria were assessed and expressed as the average ±SD (n = 3). P-values obtained from Student's unpaired two-tailed t-test,* P<0.05.
(TIF)

S13 Fig Chlamydial association with fluid phase markers and influence of EIPA on uptake. (A) Representative immunofluorescence images of cultured RPE1 cells incubated for 30 min with TRITC-dextran 70,000 MW (red) during infection of cells with *C*. *trachomatis* LGV2. Fixed cells were stained with an anti-*Chlamydia* primary antibody and an Alexa Fluor 633-conjugated secondary antibody (blue) and Alexa Fluor 488-conjugated phalloidin (green) for visualization of F-actin. Scale bars, 2.5 μm. (B) Representative immunofluorescence images of cultured RPE1 cells incubated for 30 min with TRITC-dextran 10,000 MW (red) in the presence of 10 μM EIPA in non-infected and *C*. *trachomatis* LGV2 infected cells. Fixed cells were stained with an anti-*Chlamydia* primary antibody and an Alexa Fluor 633-conjugated secondary antibody (blue). Scale bars, 10 μm.
(TIF)

S14 Fig Endogenous SNX9 and GFP-SNX9 behave as expected in cultured RPE1 cells. (A) Localisation of endogenous SNX9 and GFP-SNX9 in non-infected RPE1 cells. Cultured RPE1 cells were fixed. Fixed cells were stained with an anti-SNX9 primary antibody followed by an Alexa Fluor 488-conjugated secondary antibody. Cultured RPE1 cells were transfected with GFP-SNX9 and 18 h later were fixed. Representative images show greyscale maximum projections of confocal xy sections and inset zoom panels. Left: punctate; Right: tubular. Scale bars, 10 μm. (B) Cultured RPE1 were infected with EPEC for 4 h prior to fixation. Fixed cells were stained with an anti-SNX9 primary antibody followed by an Alexa Fluor 488-conjugated secondary antibody (green) and DRAQ5 (DNA stain, blue) to visualise the bacteria. Images show a maximum projection of confocal XY sections. Scale bar, 10 μm. (C) Cultured RPE1 cells were transfected with GFP-SNX9 and 18 h later were infected with EPEC for 4h prior to fixation. Fixed cells were stained with rhodamine-phalloidin (red) to visualise F-actin and DRAQ5 (DNA stain, blue) to visualise the bacteria. Images show a maximum projection of confocal xy sections. Scale bar, 10 μm.
(TIF)

S1 Movie Dynamic association of EBs and PI3P during *C*. *trachomatis* infection. Cultured RPE1 cells were transfected with p40-PX-GFP (PI(3)P reporter) and 18h later were infected with *C*. *trachomatis* LGV2 CMTR labelled EBs (red). Cells were incubated at 37°C for 15 min prior to imaging. Confocal z-stacks were captured every 10 s for 15 min.
(MP4)

S2 Movie GFP-SNX9 is transiently recruited during *C*. *trachomatis* infection. Cultured RPE1 cells were transfected with GFP-SNX9 and 18h later were infected with *C*. *trachomatis* LGV2 CMTR labelled EBs (red). Cells were incubated at 37°C for 15 min prior to imaging. Confocal z-stacks were captured every 49 s for 16 min.
(MP4)
