# Supplementary figures and images for: Chlamydia exploits filopodial capture and a macropinocytosis-like pathway for host cell entry
Source: PLoS Pathog. 2018 May 4;14(5):e1007051. doi: 10.1371/journal.ppat.1007051 (PMC5955597; doi:10.1371/journal.ppat.1007051)

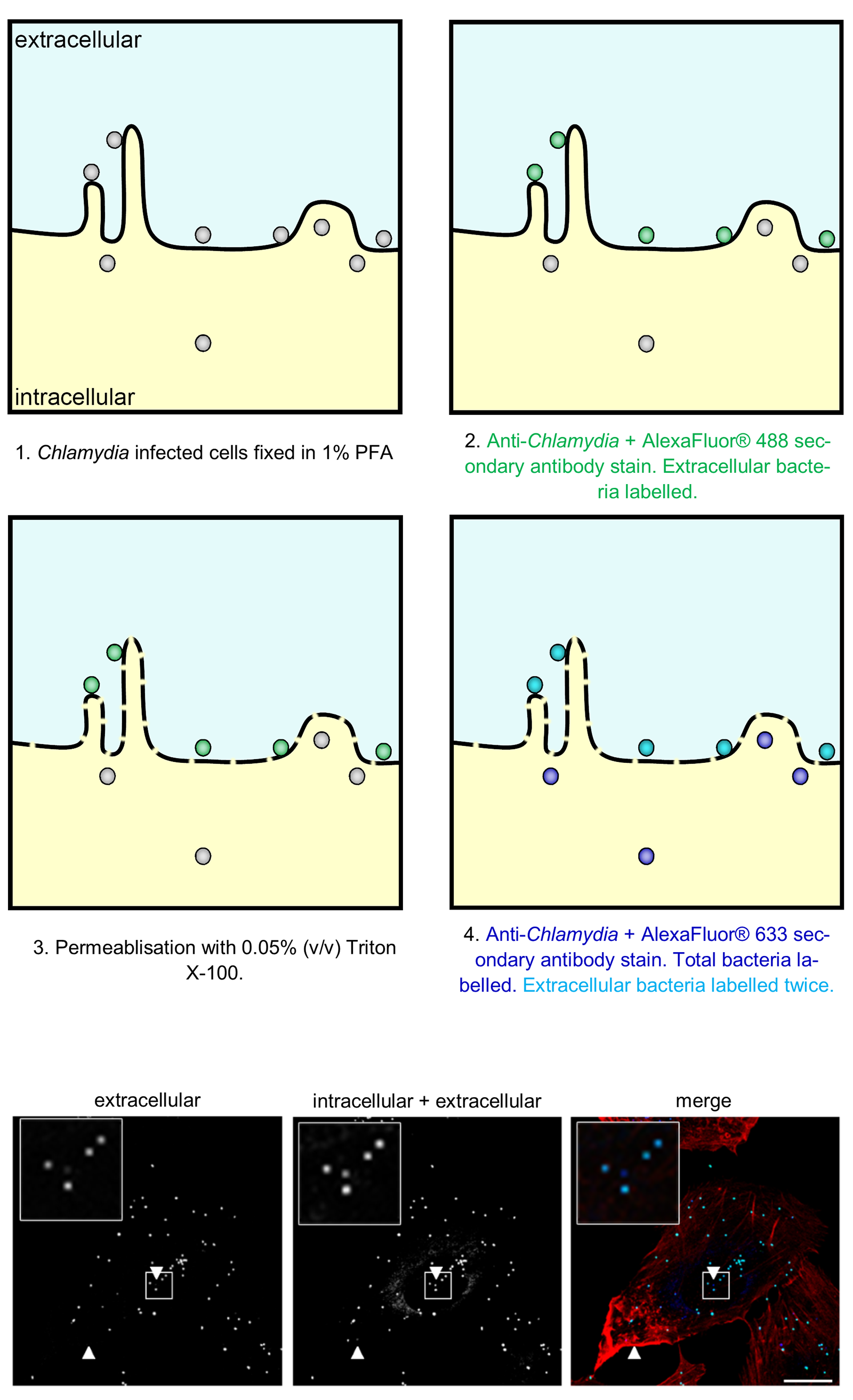

Supplement: S1 Fig — 1. Cultured cells were infected with C. trachomatis prior to fixation with 1% paraformaldehyde. 2. Fixed cells were stained with an anti-Chlamydia primary antibody and an Alexa Fluor 488-conjugated secondary antibody. 3. Cells were permeabilised with 0.05% Triton X-100 (v/v). 4. The bacteria were stained again using the same anti-Chlamydia primary antibody and an Alexa Fluor 633-conjugated secondary antibody. Intracellular bacteria were labelled with only Alexa Fluor 633 (dark blue) extracellular bacteria were labelled with Alexa Fluor 488 and Alexa Fluor 633 (green + blue, cyan; ‘extracellular’ and ‘intracellular and extracellular’ panels). Lower panels indicate an example of the resulting immunofluorescence images stained using this methodology. F-actin was stained with rhodamine-phalloidin. (TIF) [file ppat.1007051.s001.tif]

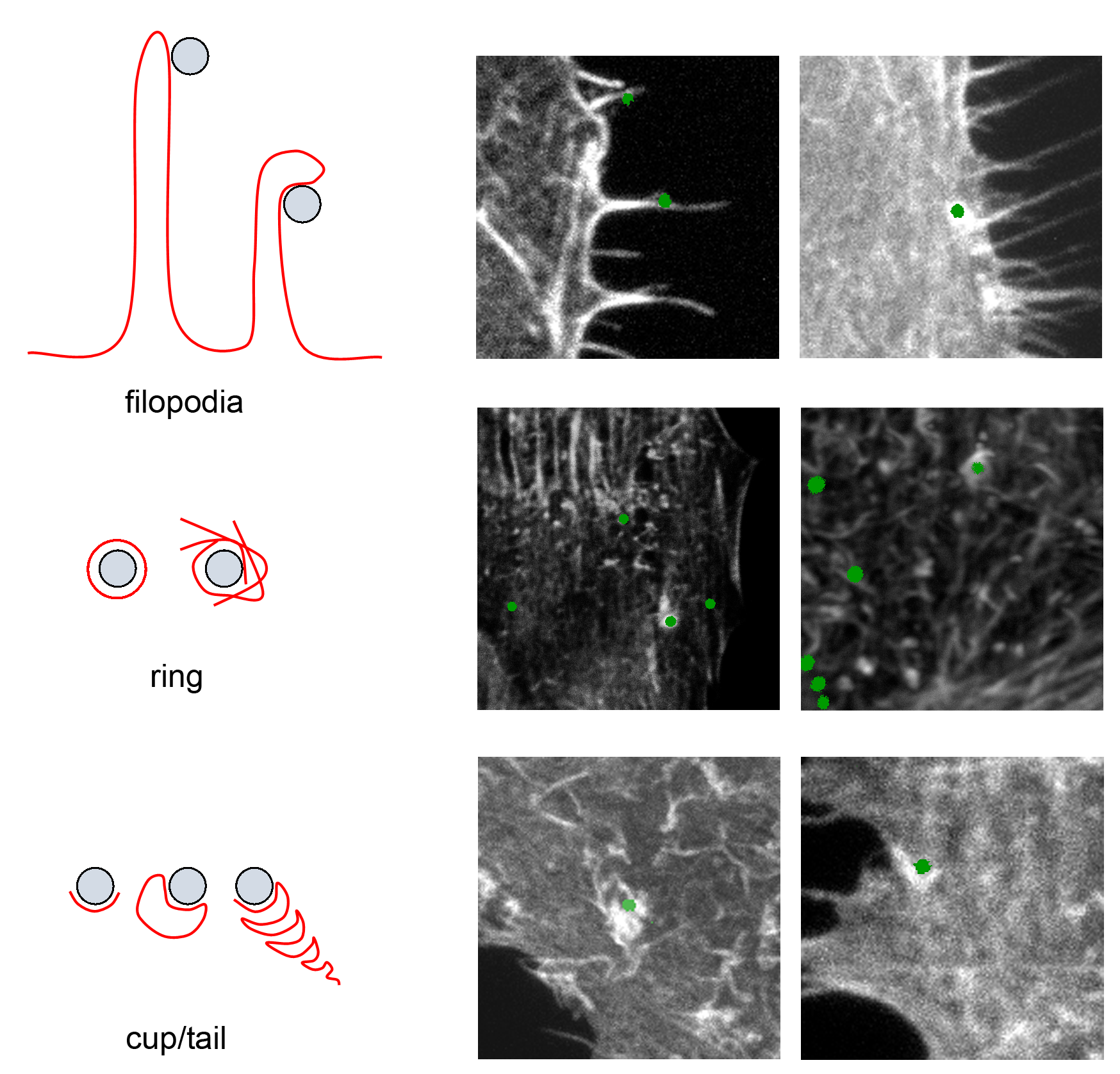

Supplement: S2 Fig — Left hand panels show diagrammatic representations of filopodia, ring and cup/tails corresponding to the F-actin structures visualised by fluorescence microscopy of cultured cells infected with C. trachomatis. Representative immunofluorescence images show cultured RPE1 cells infected with C. trachomatis LGV2 for 30 min prior to fixation. Fixed cells were stained with an anti-Chlamydia primary antibody and an Alexa Fluor 488-conjugated secondary antibody and rhodamine phalloidin. (TIF) [file ppat.1007051.s002.tif]

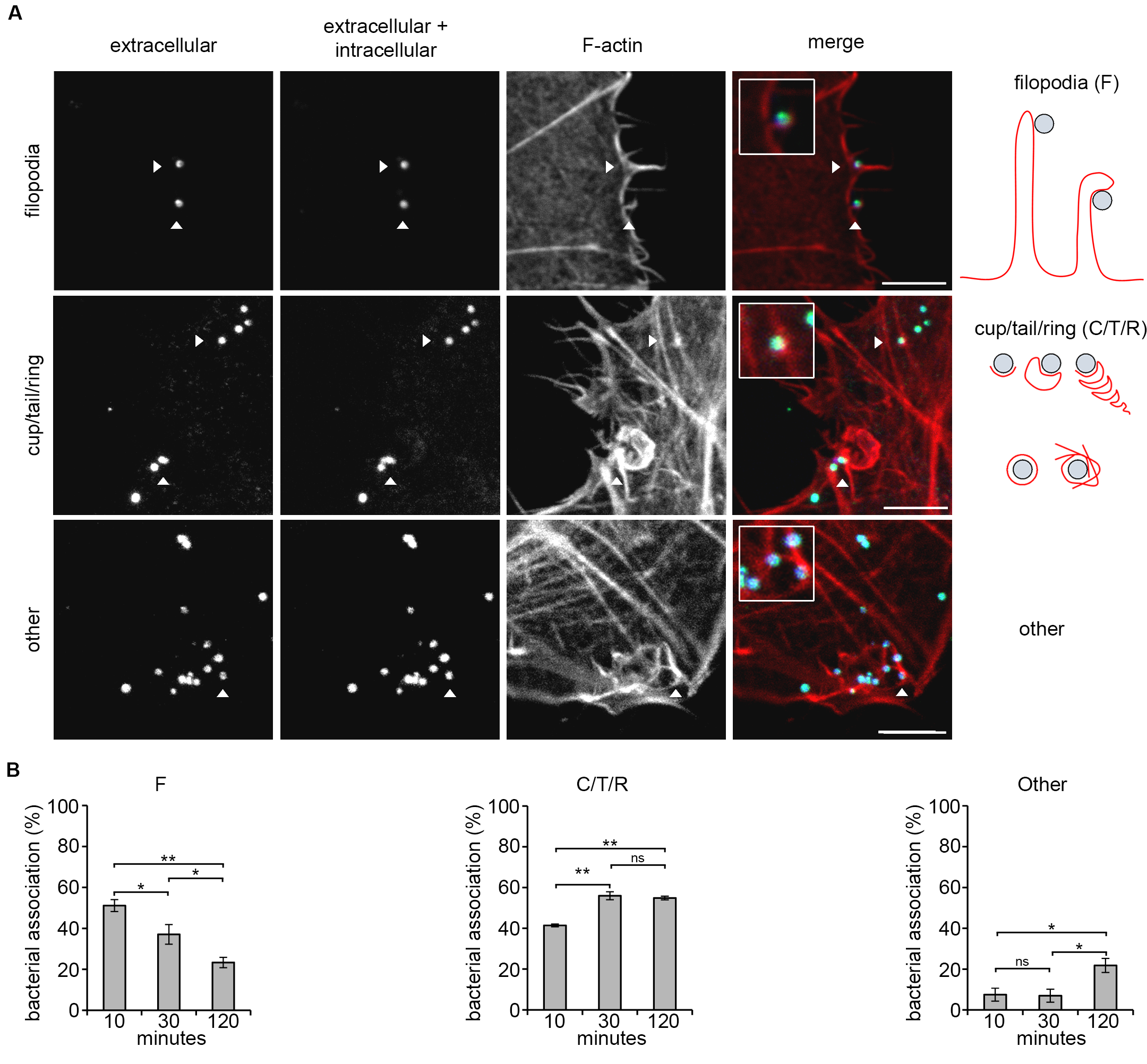

Supplement: S3 Fig — (A) Representative immunofluorescence images of F-actin recruitment to C. trachomatis EBs during early interaction with HeLa cells. Cultured HeLa cells were infected with C. trachomatis LGV2 for 30 minutes prior to fixation with 1% paraformaldehyde. Fixed cells were stained with an anti-Chlamydia primary antibody and an Alexa Fluor 488-conjugated secondary antibody. Cells were permeabilised with 0.05% Triton X-100 (v/v) and the bacteria stained using the same anti-Chlamydia primary antibody and an Alexa Fluor 633-conjugated secondary antibody. Intracellular bacteria were labelled with only Alexa Fluor 633 (dark blue; ‘intracellular and extracellular’ panel), extracellular bacteria were labelled with Alexa Fluor 488 and Alexa Fluor 633 (green + blue, cyan; ‘extracellular’ and ‘intracellular and extracellular’ panels). F-actin was stained with rhodamine-phalloidin. White arrowheads show typical examples of indicated classes of F-actin structure. Images are maximum projections of confocal xy sections. Scale bars, 5 μm. Right hand panels show diagrammatic representations of the defined classes of F-actin structures visualised by fluorescence microscopy of cultured HeLa cells infected with C. trachomatis. (B) Quantification of F-actin structures associated with extracellular C. trachomatis EBs from 10–120 min post-infection of HeLa cells. Cultured HeLa cells were infected with C. trachomatis LGV2 for 10, 30, and 120 min prior to fixation with 1% paraformaldehyde. Fixed cells were stained as above and the association of EBs with the defined F-actin classes was quantified. ≥ 200 bacteria were assessed at each time point and the percentage of EBs in association with each class of structure was calculated, expressed as the average ±SD (n = 3). ≥ 200 bacteria were assessed at each time point and the percentage of EBs in association with each class of structure was calculated, expressed as the average ±SD (n = 3). * P<0.05, ** P<0.01, ‘ns’ not significant using one-way ANOVA f [file ppat.1007051.s003.tif]

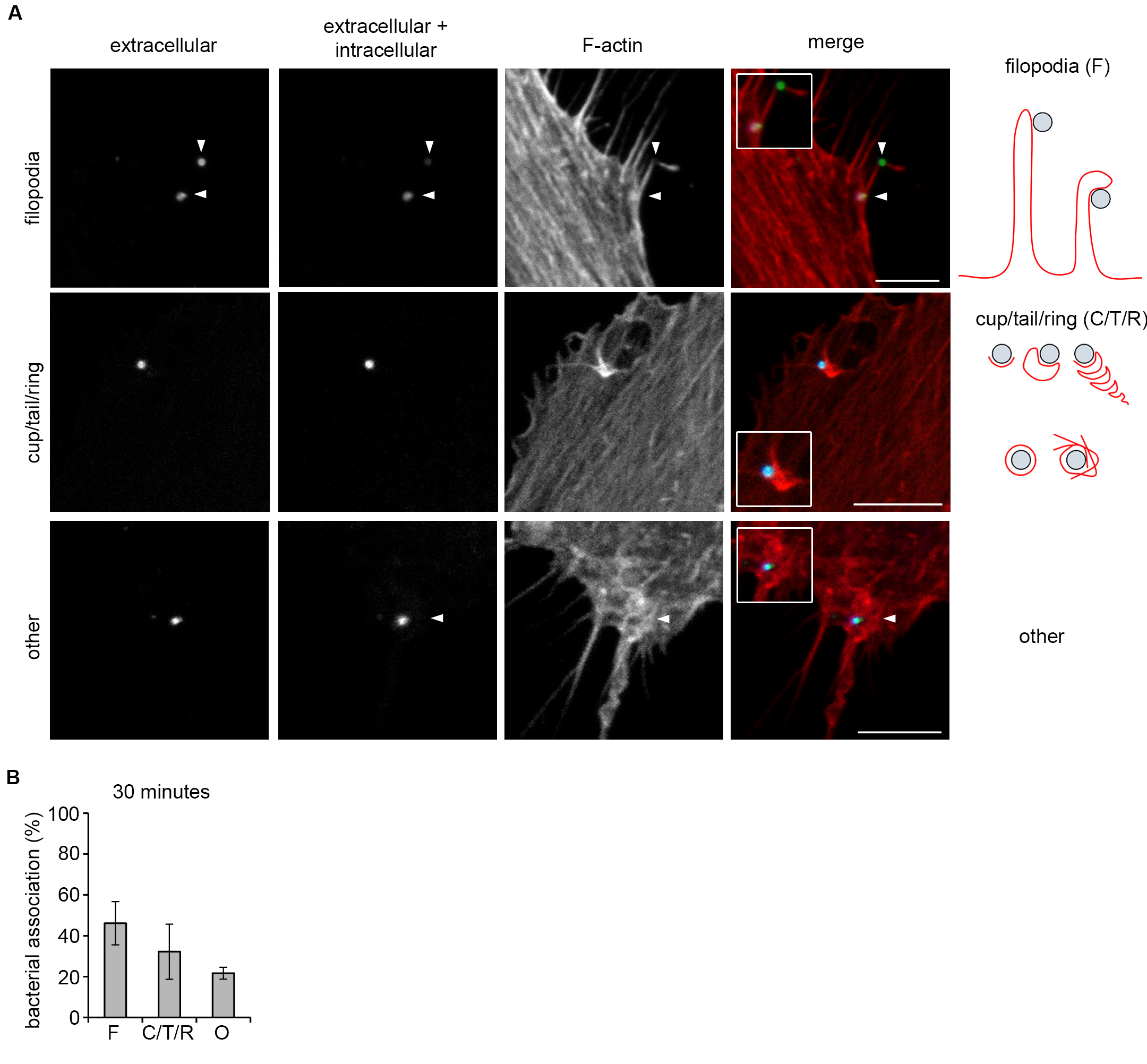

Supplement: S4 Fig — (A) Representative immunofluorescence images of F-actin recruitment to C. trachomatis serovar D EBs during early interaction with RPE1 cells. Cultured RPE1 cells were infected with C. trachomatis LGV2 prior to fixation with 1% paraformaldehyde. Fixed cells were stained with an anti-Chlamydia primary antibody and an Alexa Fluor 488-conjugated secondary antibody. Cells were permeabilised with 0.05% Triton X-100 (v/v) and the bacteria stained using the same anti-Chlamydia primary antibody and an Alexa Fluor 633-conjugated secondary antibody. Intracellular bacteria were labelled with only Alexa Fluor 633 (dark blue; ‘intracellular and extracellular’ panel), extracellular bacteria were labelled with Alexa Fluor 488 and Alexa Fluor 633 (green + blue, cyan; ‘extracellular’ and ‘intracellular and extracellular’ panels). F-actin was stained with rhodamine-phalloidin. White arrowheads show typical examples of indicated classes of F-actin structure. Images are maximum projections of confocal xy sections. Scale bars, 5 μm. Right hand panels show diagrammatic representations of the defined classes of F-actin structures visualised by fluorescence microscopy of cultured RPE1 cells infected with C. trachomatis serovar D. (B) Quantification of F-actin structures associated with extracellular C. trachomatis EBs at 30 min post-infection of RPE1 cells. Cultured RPE1 cells were infected with C. trachomatis serovar D for 30 min prior to fixation with 1% PFA. Fixed cells were stained as above and the association of EBs with the defined F-actin classes was quantified. ≥ 200 bacteria were assessed and the percentage of EBs in association with each class of structure was calculated, expressed as the average ±SD (n = 3). (TIF) [file ppat.1007051.s004.tif]

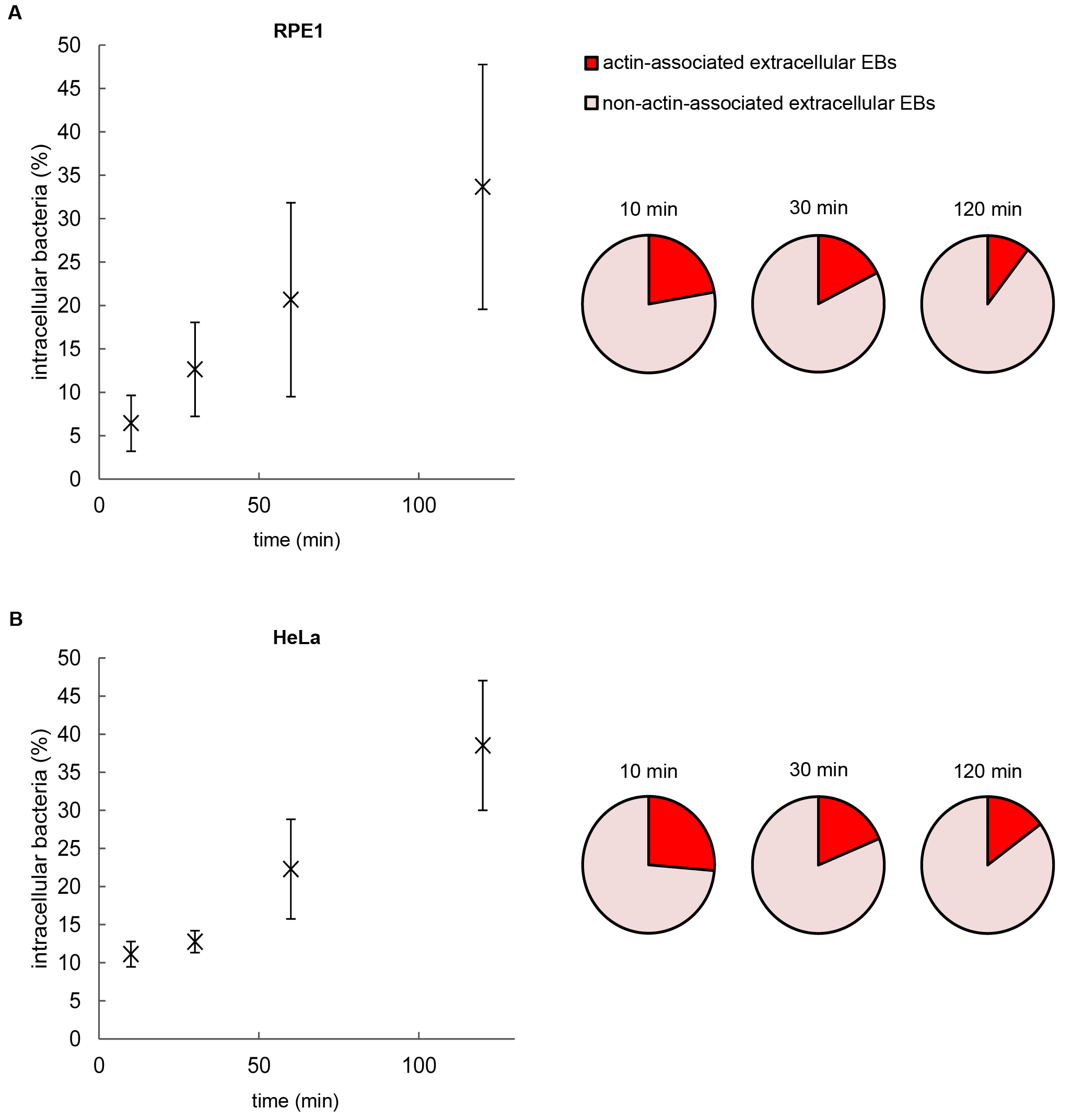

Supplement: S5 Fig — (A) Cultured RPE1 cells were infected with C. trachomatis LGV2 for the indicated timepoints prior to fixation with 1% paraformaldehyde. Fixed cells were stained with an anti-Chlamydia primary antibody and an Alexa Fluor 488-conjugated secondary antibody. Cells were permeabilised with 0.5% Triton X-100 (v/v) and the bacteria stained using the same anti-Chlamydia primary antibody and an Alexa Fluor 633-conjugated secondary antibody. Intracellular bacteria were labelled with only Alexa Fluor 633, extracellular bacteria were labelled with Alexa Fluor 488 and Alexa Fluor 633. The numbers of intracellular bacteria at each time point was quantified. ≥ 300 bacteria were assessed at each time point and the percentage of intracellular bacteria is expressed as the average ±SD (n = 3). Right hand pie charts indicate the average percentage of F-actin associated extracellular EBs at the indicated timepoint. (B) Cultured HeLa cells were infected with C. trachomatis LGV2 for the indicated timepoints prior to fixation with 1% paraformaldehyde. Fixed cells were stained with an anti-Chlamydia primary antibody and an Alexa Fluor 488-conjugated secondary antibody. Cells were permeabilised with 0.5% Triton X-100 (v/v) and the bacteria stained using the same anti-Chlamydia primary antibody and an Alexa Fluor 633-conjugated secondary antibody. Intracellular bacteria were labelled with only Alexa Fluor 633, extracellular bacteria were labelled with Alexa Fluor 488 and Alexa Fluor 633. The numbers of intracellular bacteria at each time point was quantified. ≥ 300 bacteria were assessed at each time point and the percentage of intracellular bacteria is expressed as the average ±SD (n = 3). Right hand pie charts indicate the average percentage of F-actin associated extracellular EBs at the indicated timepoint. (TIF) [file ppat.1007051.s005.tif]

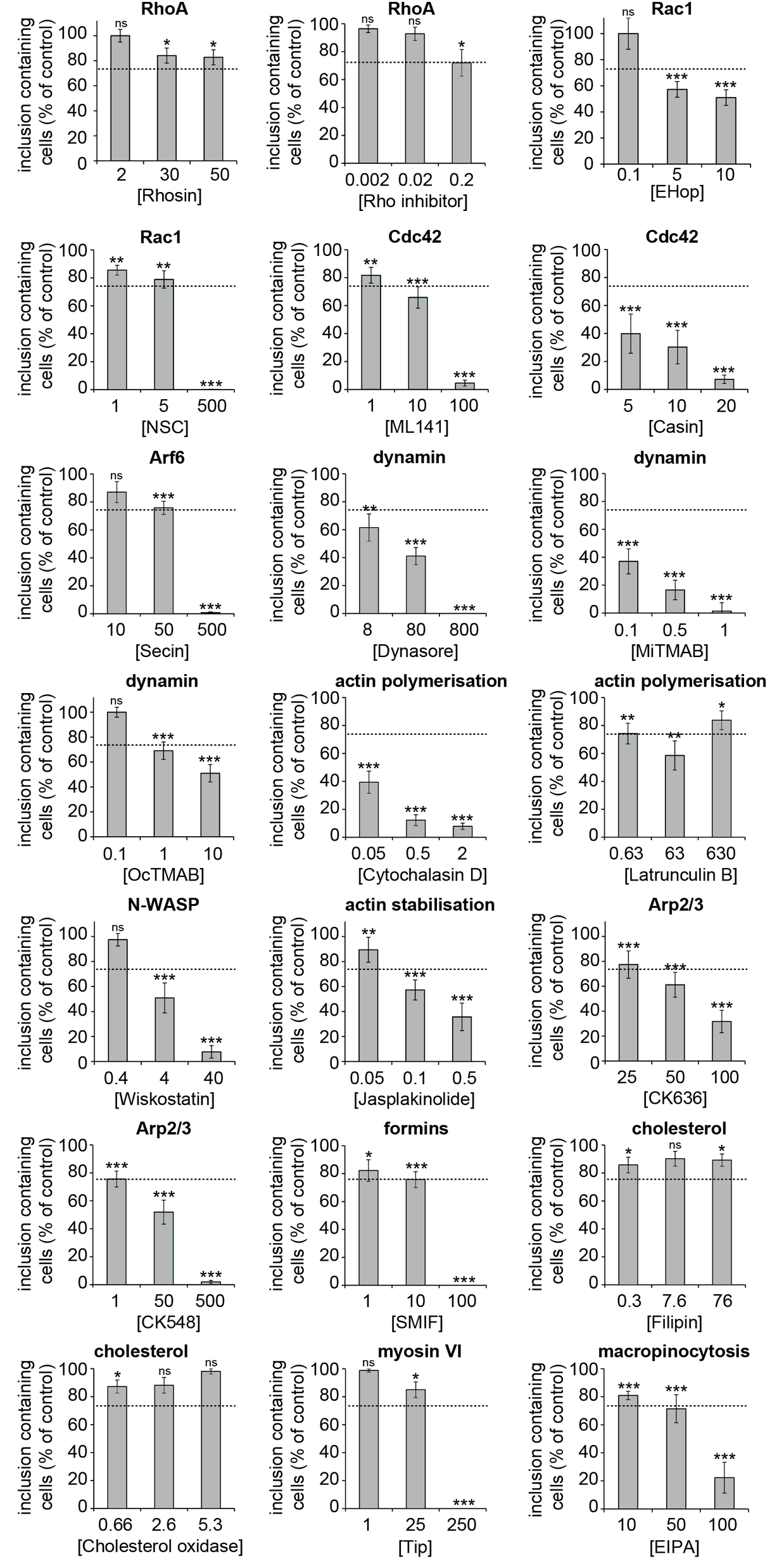

Supplement: S6 Fig — Cultured RPE1 cells were pre-treated for 5 min with the indicated concentration of the inhibitor, followed by infection with Chlamydia trachomatis LGV2 in the presence of inhibitor. At 2 h post-infection the inhibitor was washed out and infection was allowed to progress for a further 22 h (i.e. until 24 h post-infection). Cells were then fixed and stained with an anti-Chlamydia primary antibody and an Alexa Fluor 488-conjugated secondary antibody. The average number of inclusion-containing cells was quantified for 10 fields of view in both inhibitor and mock treated samples at the three indicated concentrations (μM), expressed as the relative percentage of inclusion containing cells ±SEM (n = 2). A dotted line is drawn at ≥ 75%, indicating where inhibitors decreased inclusion formation by ≥ 25% of control cells. P-values obtained from Student's unpaired two-tailed t-test, * P<0.05, ** P<0.01, *** P<0.001, ‘ns’ not significant. (TIF) [file ppat.1007051.s006.tif]

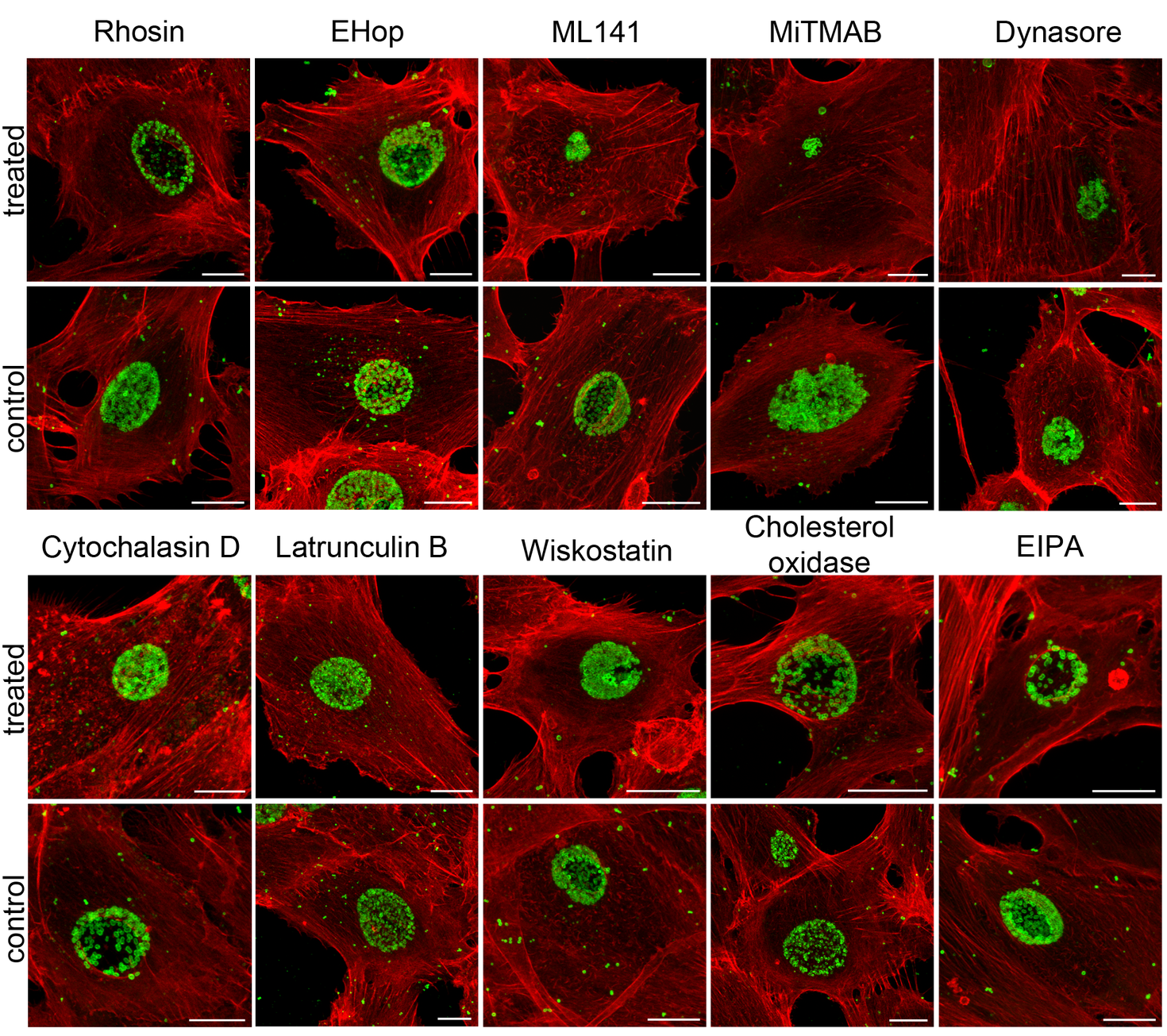

Supplement: S7 Fig — Cultured RPE1 cells were pre-treated for 5 min with the mid concentration of the indicated inhibitor, followed by infection with Chlamydia trachomatis LGV2 in the presence of inhibitor (or equivalent mock control treatments). At 2 h post-infection the inhibitor was washed out and infection was allowed to progress for a further 22 h (i.e. until 24 h post-infection). Cells were then fixed and stained with an anti-Chlamydia primary antibody and an Alexa Fluor 488-conjugated secondary antibody (green) and rhodamine-phalloidin (red). Images are maximum projections of confocal xy sections. Scale bars, 10 μm. (TIF) [file ppat.1007051.s007.tif]

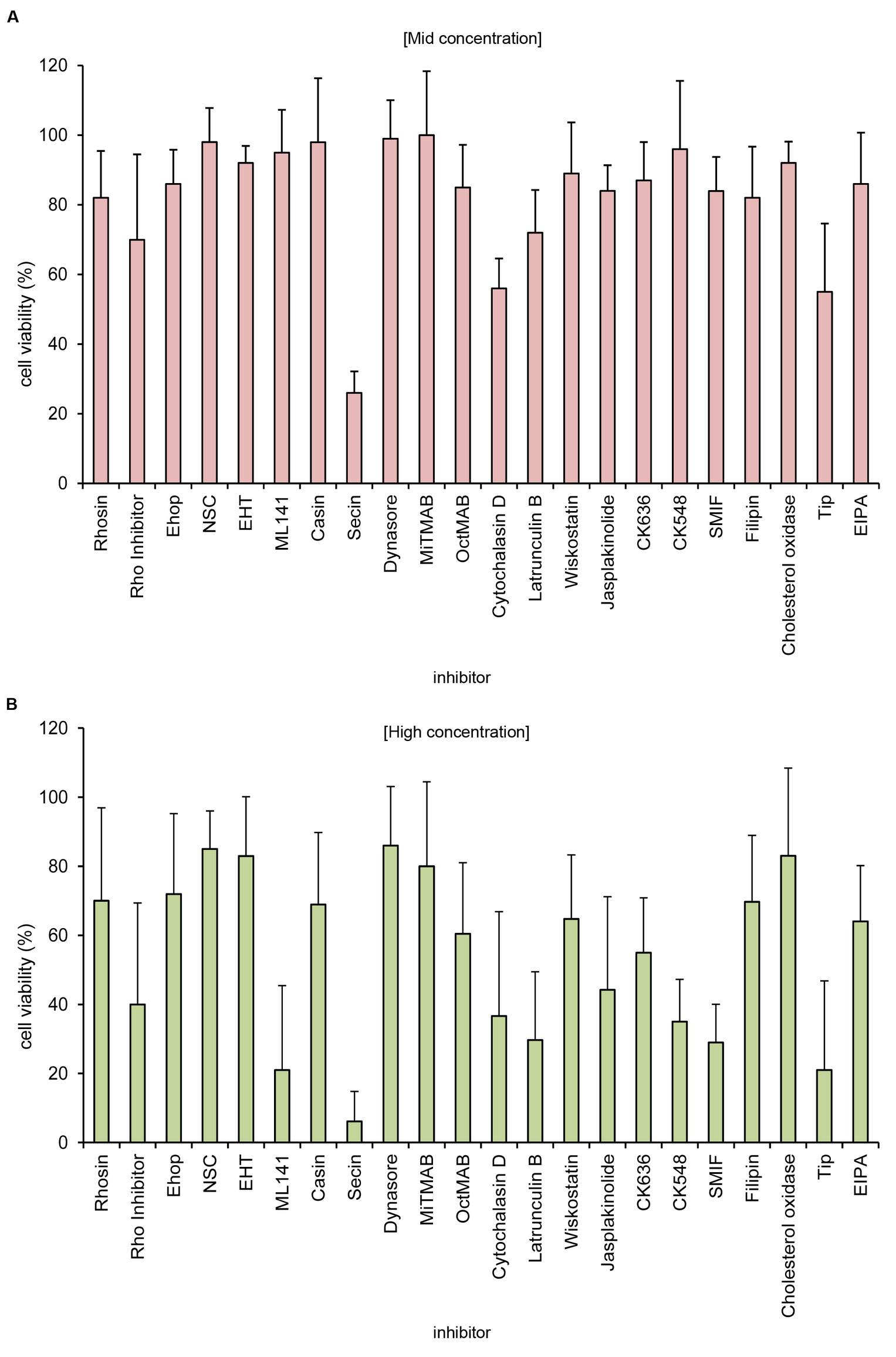

Supplement: S8 Fig — (A) and (B) Cultured RPE1 cells were treated for 2 h with either the mid or high concentration of the indicated inhibitor (see S3 Fig). Viable cells (non-permeable to trypan blue) were enumerated for inhibitor and mock-treated cells (n = 2) and the percentage viable cells relative to mock-treated control cells was calculated. (TIF) [file ppat.1007051.s008.tif]

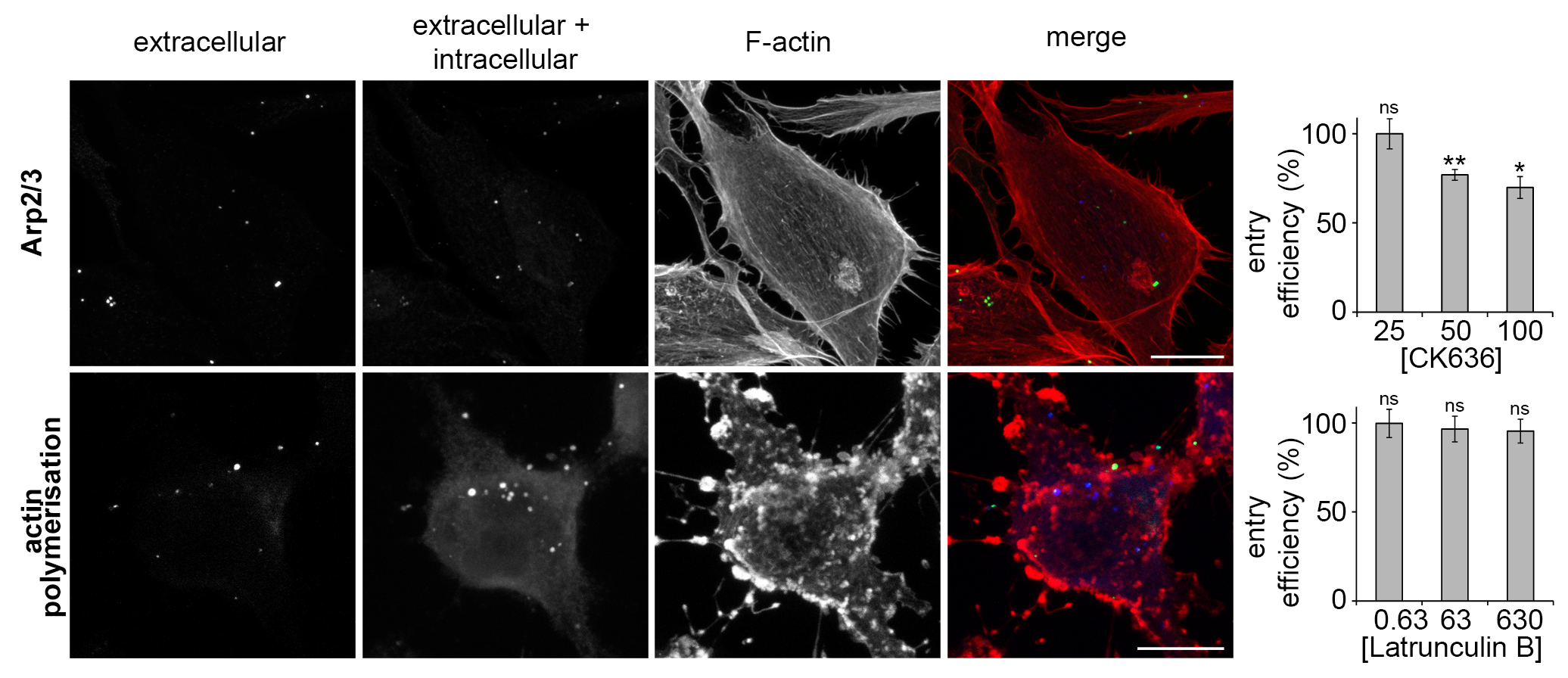

Supplement: S9 Fig — Cultured RPE1 cells were pre-treated for 5 min with the indicated concentration of the inhibitor, followed by infection with Chlamydia trachomatis LGV2 in the presence of inhibitor. At 2 h post-infection the cells were washed then fixed with 1% PFA and stained with an anti-Chlamydia primary antibody and an Alexa Fluor 488-conjugated secondary antibody (green). Cells were permeabilised with 0.05% Triton X-100 (v/v) and the bacteria stained using the same anti-Chlamydia primary antibody and an Alexa Fluor 633-conjugated secondary antibody. Intracellular bacteria were labelled with only Alexa Fluor 633 (dark blue; ‘intracellular and extracellular’ panel), extracellular bacteria were labelled with Alexa Fluor 488 and Alexa Fluor 633 (green + blue, cyan; ‘extracellular’ and ‘intracellular and extracellular’ panels). Rhodamine phalloidin (red) visualises F-actin. Images are maximum projections of confocal xy sections. Scale bars, 10 μm. The entry efficiency in the presence of inhibitors relative to mock treated control cells was quantified. ≥ 300 bacteria were assessed at each time point and the percentage entry efficiency of EBs is expressed as the average ±SD (n = 3). P-values obtained from Student's unpaired two-tailed t-test, * P<0.05, ** P<0.01, ‘ns’ not significant. (TIF) [file ppat.1007051.s009.tif]

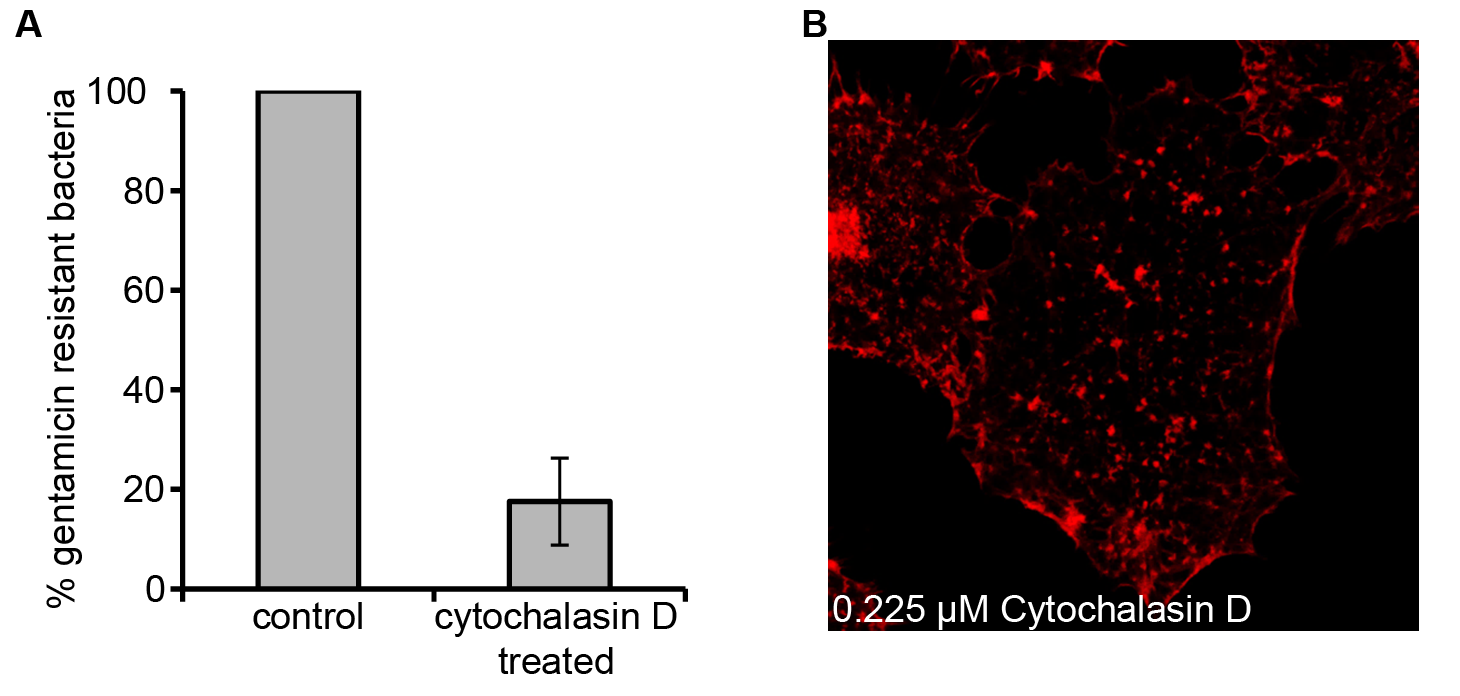

Supplement: S10 Fig — (A) RPE1 cells were pre-treated for 5 minutes with 2 μM cytochalasin D and infected with Salmonella enterica serovar typhimurium SL1344 in infection medium containing cytochalasin D. Infection was allowed to proceed for 1 h before cells were washed and further incubated for 1 h in gentamicin-containing cell culture medium. Cells were then lysed and serial dilutions of the resulting lysate were plated onto solid agar. The number of colonies recovered from cytochalasin D and mock-treated control cells was quantified. Results are expressed as a percentage relative to mock treated control ±SD (n = 3). (B) Non-infected RPE1 cells treated with cytochalasin D for 1 h at the indicated concentration. Cells were then fixed and stained with rhodamine-phalloidin to visualize F-actin. (TIF) [file ppat.1007051.s010.tif]

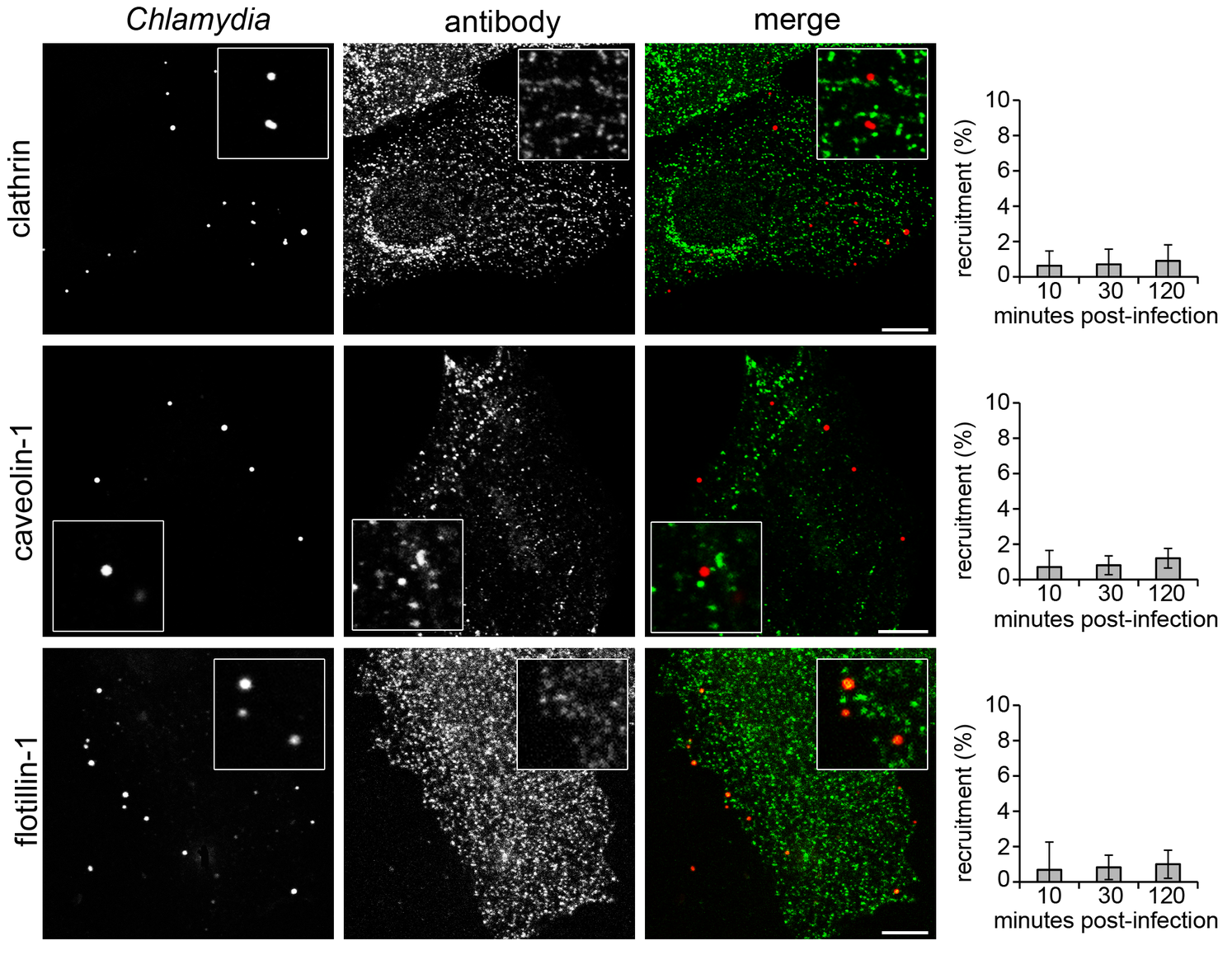

Supplement: S11 Fig — Cultured RPE1 cells were infected with C. trachomatis LGV2 for 10, 30 or 120 min prior to fixation. Fixed cells were stained with an anti-Chlamydia primary antibody and an Alexa Fluor 546-conjugated secondary antibody (red) and primary antibodies corresponding to clathrin, caveolin-1 or flotillin-1 followed by an Alexa Fluor 488-conjugated secondary antibody (green). Representative images are from 30 min post-infection and show the whole cell with a close-up of an EB-containing region and are maximum projections of confocal xy sections of the focal plane containing the EB and the nearest neighbour z-sections (±0.2 μm). Scale bars, 10 μm. ≥ 100 cell-associated bacteria were assessed for recruitment of the markers during entry, expressed as the average ±SD (n = 3). (TIF) [file ppat.1007051.s011.tif]

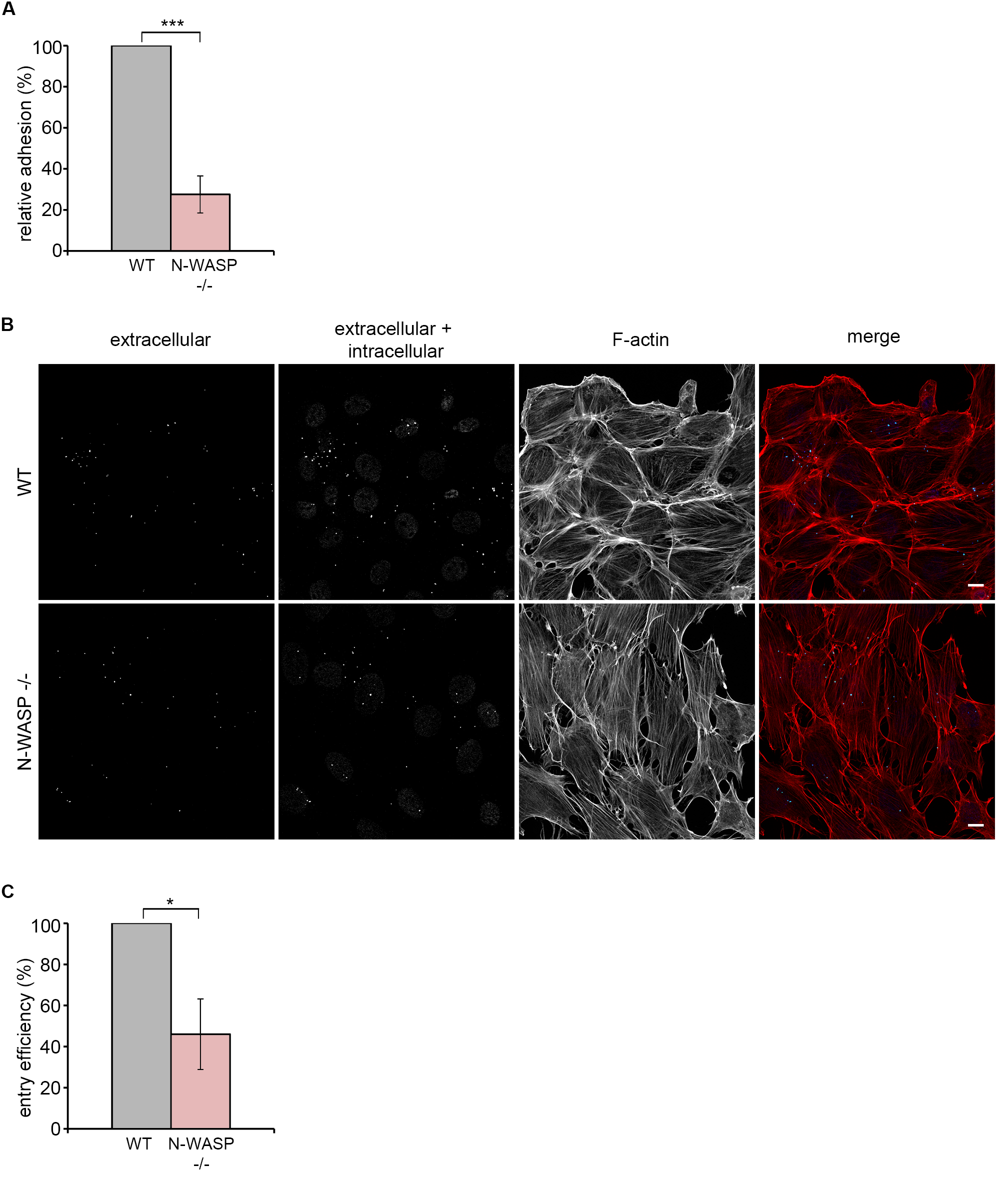

Supplement: S12 Fig — (A) Adhesion is greatly attenuated in N-WASP-/- mouse embryonic fibroblasts. Numbers of adherent bacteria per cell were quantified following an adhesion assay. N-WASP knockout MEFs were incubated at 4°C for 1 h with infection medium containing C. trachomatis LGV2 in suspension at an MOI of 100. Cells were washed and fixed. Fixed cells were stained with an anti-Chlamydia primary antibody and an AlexaFluor 488-conjugated secondary antibody and the number of adherent bacteria per cell was assessed for ≥ 25 cells, expressed as the average ±SD (n = 3). P-values obtained from Student's unpaired two-tailed t-test, *** P<0.001. (B) Entry efficiency is decreased in N-WASP knockout cells. Immunofluorescence examples of cultured WT and N-WASP-/- mouse embryonic fibroblasts infected with C. trachomatis LGV2. At 2 h post-infection the cells were washed then fixed with 1% PFA and stained with an anti-Chlamydia primary antibody and an Alexa Fluor 488-conjugated secondary antibody. Cells were permeablised and stained again using anti-Chlamydia primary antibody and an Alexa Fluor 633-conjugated secondary antibody. Intracellular bacteria were labelled with only one fluorophore (Alexa Fluor 633). Scale bars, 10 μm. (C) Cells were infected and stained as above. Entry efficiency was calculated in N-WASP-/- cells relative to WT cells. ≥ 200 cell-associated bacteria were assessed and expressed as the average ±SD (n = 3). P-values obtained from Student's unpaired two-tailed t-test,* P<0.05. (TIF) [file ppat.1007051.s012.tif]

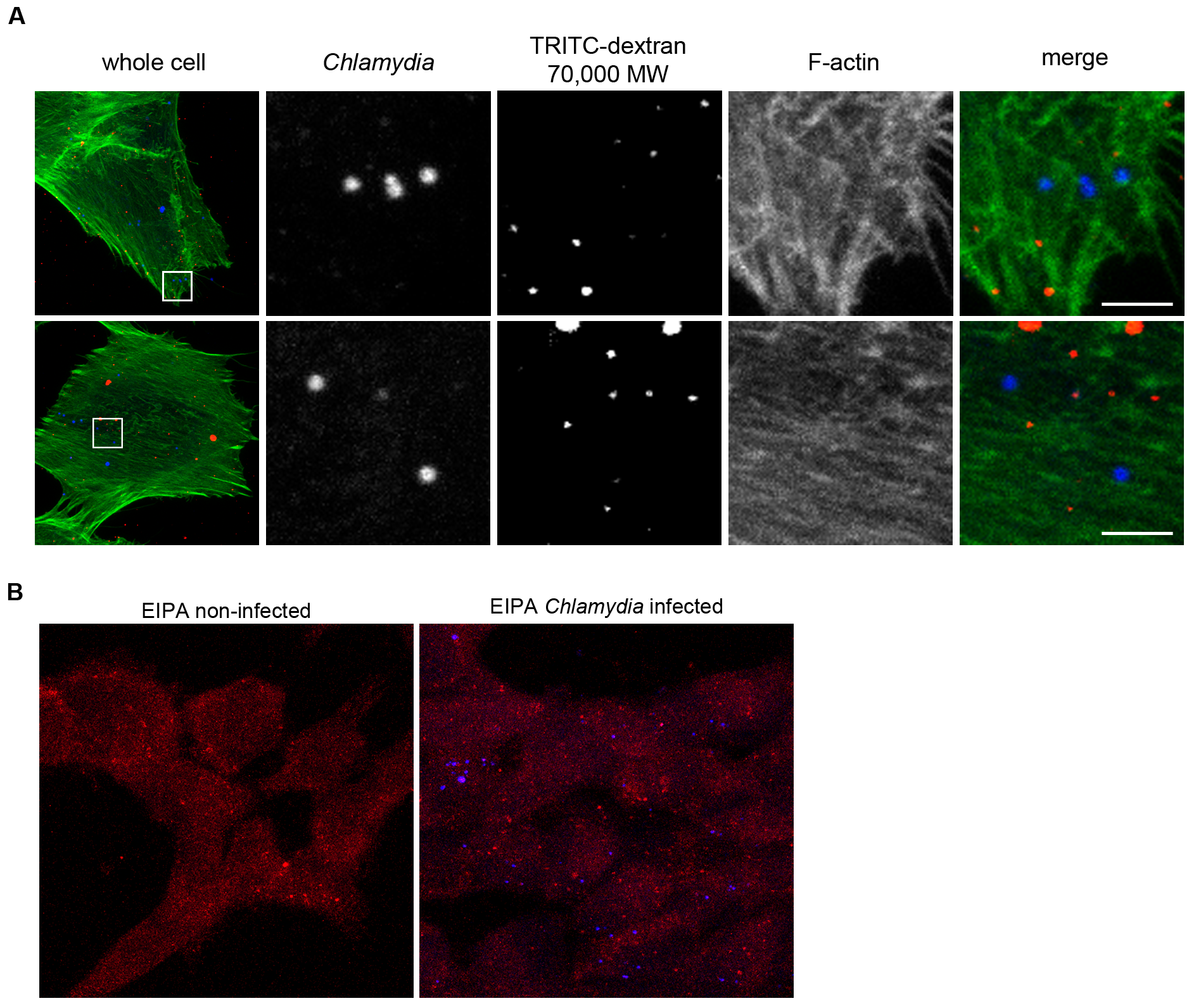

Supplement: S13 Fig — (A) Representative immunofluorescence images of cultured RPE1 cells incubated for 30 min with TRITC-dextran 70,000 MW (red) during infection of cells with C. trachomatis LGV2. Fixed cells were stained with an anti-Chlamydia primary antibody and an Alexa Fluor 633-conjugated secondary antibody (blue) and Alexa Fluor 488-conjugated phalloidin (green) for visualization of F-actin. Scale bars, 2.5 μm. (B) Representative immunofluorescence images of cultured RPE1 cells incubated for 30 min with TRITC-dextran 10,000 MW (red) in the presence of 10 μM EIPA in non-infected and C. trachomatis LGV2 infected cells. Fixed cells were stained with an anti-Chlamydia primary antibody and an Alexa Fluor 633-conjugated secondary antibody (blue). Scale bars, 10 μm. (TIF) [file ppat.1007051.s013.tif]

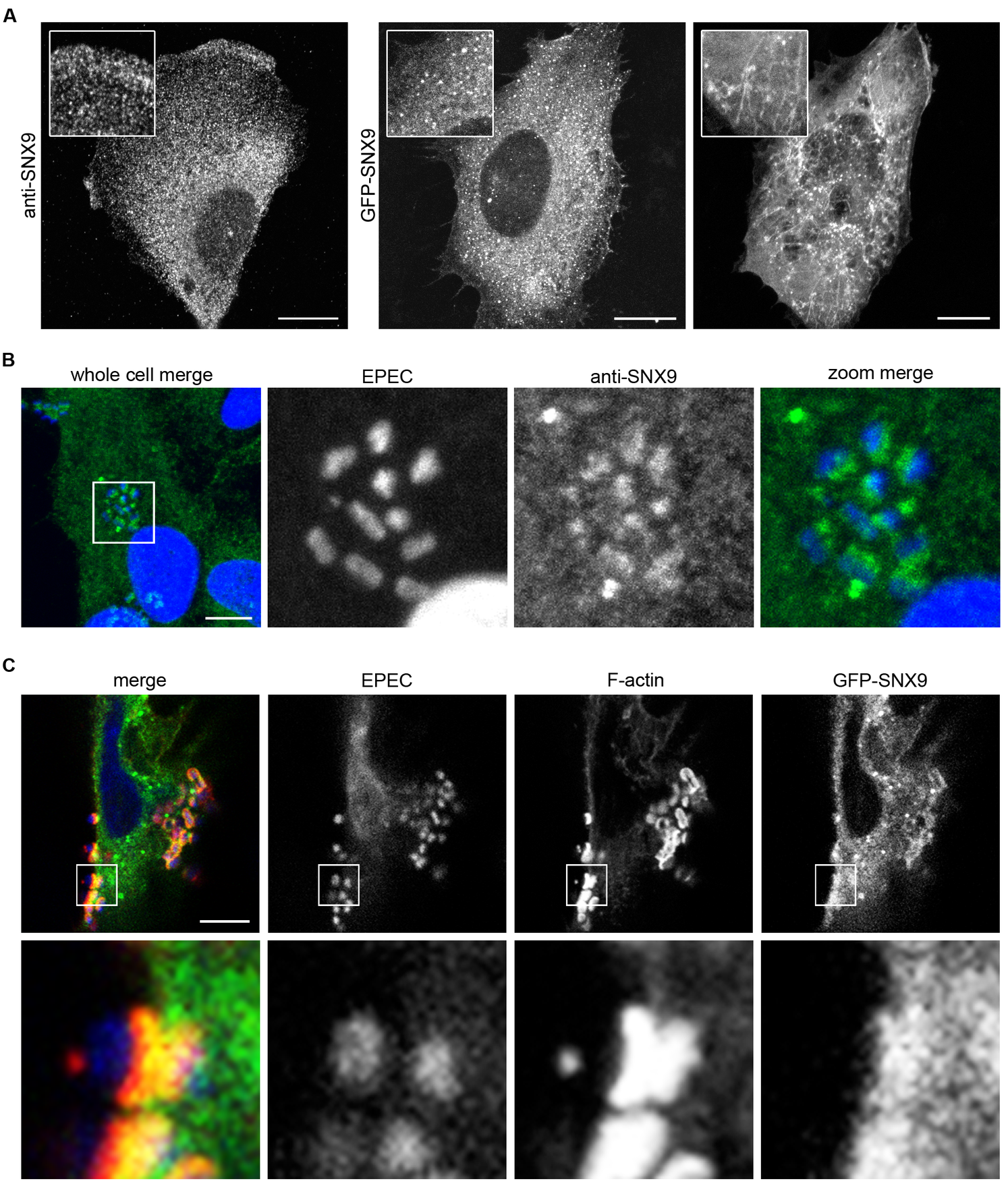

Supplement: S14 Fig — (A) Localisation of endogenous SNX9 and GFP-SNX9 in non-infected RPE1 cells. Cultured RPE1 cells were fixed. Fixed cells were stained with an anti-SNX9 primary antibody followed by an Alexa Fluor 488-conjugated secondary antibody. Cultured RPE1 cells were transfected with GFP-SNX9 and 18 h later were fixed. Representative images show greyscale maximum projections of confocal xy sections and inset zoom panels. Left: punctate; Right: tubular. Scale bars, 10 μm. (B) Cultured RPE1 were infected with EPEC for 4 h prior to fixation. Fixed cells were stained with an anti-SNX9 primary antibody followed by an Alexa Fluor 488-conjugated secondary antibody (green) and DRAQ5 (DNA stain, blue) to visualise the bacteria. Images show a maximum projection of confocal XY sections. Scale bar, 10 μm. (C) Cultured RPE1 cells were transfected with GFP-SNX9 and 18 h later were infected with EPEC for 4h prior to fixation. Fixed cells were stained with rhodamine-phalloidin (red) to visualise F-actin and DRAQ5 (DNA stain, blue) to visualise the bacteria. Images show a maximum projection of confocal xy sections. Scale bar, 10 μm. (TIF) [file ppat.1007051.s014.tif]
